# Mapping the Young‐Onset Dementia Research in the Asia‐Pacific Region: A Scoping Review

**DOI:** 10.1111/appy.70030

**Published:** 2026-07-14

**Authors:** Gia Tan, Gary Cheung, Samantha M. Loi, Etuini Ma'u, Campbell Le Heron, Monica Cations, Nick Garrett, Megan Eustace, So Young Moon, Eun‐Joo Kim, Kok Pin Ng, Adeline S. L. Ng, Huali Wang, Isabelle Burke, Kenny Ong, Brigid Ryan

**Affiliations:** ^1^ Department of Psychological Medicine, School of Medicine, Faculty of Medical and Health Science University of Auckland Auckland New Zealand; ^2^ Department of Psychiatry University of Melbourne Melbourne Victoria Australia; ^3^ Neuropsychiatry Centre Royal Melbourne Hospital Parkville Victoria Australia; ^4^ New Zealand Brain Research Institute Christchurch New Zealand; ^5^ Department of Medicine University of Otago Christchurch New Zealand; ^6^ Department of Neurology Christchurch Hospital, Te Whatu Ora Waitaha Canterbury Canterbury New Zealand; ^7^ College of Education, Psychology and Social Work Flinders University Adelaide Australia; ^8^ Biostatistics and Epidemiology Department Auckland University of Technology Auckland New Zealand; ^9^ School of Psychology, Speech and Hearing University of Canterbury Christchurch New Zealand; ^10^ Department of Neurology Ajou University School of Medicine Suwon Republic of Korea; ^11^ Department of Neurology Pusan National University Hospital, Pusan National University School of Medicine and Medical Research Institute Busan Republic of Korea; ^12^ Department of Neurology National Neuroscience Institute Singapore; ^13^ Dementia Care and Research Center Peking University Institute of Mental Health (Sixth Hospital), National Clinical Research Center for Mental Disorders Beijing China; ^14^ School of Psychology, Faculty of Health Deakin University Burwood Victoria Australia; ^15^ Neuropsychiatry Unit, Department of Psychiatry & Mental Health, Tunku Abdul Rahman Institute of Neuroscience Hospital Kuala Lumpur Kuala Lumpur Malaysia; ^16^ Department of Anatomy and Medical Imaging, Faculty of Medical and Health Science University of Auckland Auckland New Zealand; ^17^ Centre for Brain Research, Faculty of Medical and Health Science University of Auckland Auckland New Zealand

**Keywords:** Asia‐Pacific, neuropsychiatry, research, review, young‐onset dementia

## Abstract

Young‐onset dementia (YOD), with symptom onset before 65, is an area of increasing public health importance. YOD research in Asia‐Pacific remains under‐represented in the global YOD research landscape. This scoping review aimed to comprehensively map the existing YOD literature from Asia‐Pacific and provide an overview of the research topics explored to date. We followed the PRISMA extension for scoping reviews guidelines and searched Scopus and PsycINFO, using predefined search terms, from their inception to December 31, 2024. Empirical studies involving participants with YOD, or family carers of people with YOD, living in Asia‐Pacific, were included. 437 publications were identified. Fourteen of the 42 Asia‐Pacific countries/territories have published YOD research, with Japan, Australia, South Korea, and China contributing 82.2% of YOD research in the region. The volume of YOD publications in the Asia‐Pacific region increased steadily from the early 2010s. Most studies were case reports/series (34.8%) and quantitative (59.2%), with 5.9% using qualitative or mixed‐methods. The YOD research landscape is mostly focused on disease biology such as genetics, neuroimaging, and neuropathology. Despite the impacts of YOD on individuals and their families, only 11.4% of publications, mainly from Australia, examined psychosocial impacts and support services. There is a need for research in the Asia–Pacific region to better understand the journey of YOD and to co‐create interventions and services that address the needs of those affected. Potential policy implications include explicit inclusion of YOD within national and regional dementia policies, with acknowledgement that psychosocial impacts of YOD are insufficiently addressed by older age‐based dementia frameworks.

## 1 Background

Young‐onset dementia (YOD) is defined as dementia with symptom onset before the age of 65 (van de Veen et al. 2022). Over the past decade, there has been growing recognition of the distinct diagnostic and postdiagnostic challenges faced by people with YOD and their families, which differ from those experienced by older adults with dementia; individuals affected are often employed, have financial commitments, and are caring for children (Luscombe et al. 1998; McMurtray et al. 2006; Ryan et al. 2021; van Vliet et al. 2010; van Vliet et al. 2013; Williams et al. 2001). The specific needs of people living with YOD are increasingly being recognized, with the whole family affected, and clinicians are urged to improve care for this group (Cations et al. 2021).

YOD is a heterogenous neurodegenerative condition with diverse presenting symptoms across cognitive, behavioral, psychiatric, and neurological domains (Draper and Withall 2016). Most studies report that Alzheimer's disease is the most common cause of YOD, followed by vascular dementia and frontotemporal dementia (Hendriks et al. 2021). These three dementia subtypes account for 50%–75% of all YOD cases (Vieira et al. 2013), although it is likely that Dementia with Lewy bodies also makes a significant contribution (Kane et al. 2018). Globally, YOD has been estimated to affect approximately 3.9 million people. A previous meta‐analysis suggested a global prevalence of 119 per 100 000 population aged 30–64 (Hendriks et al. 2021). However, a more recent study reported considerably higher global YOD age‐specific prevalence estimates with over 300 per 100 000 population (Li et al. 2024).

YOD research has seen significant growth in the past decade, driven by advances in identifying underlying etiologies, understanding progression, and exploring psychosocial dimensions, including the needs and preferences of individuals with YOD and their families. While substantial research has been conducted in Western countries, the Asia‐Pacific region remains underrepresented in the global YOD research landscape, despite accounting for half of the world's population (Alzheimer's Disease International 2014). There are also unique challenges relating to dementia in the Asia‐Pacific region, including limited awareness and stigma of dementia, cultural interpretations of aging, limited human and financial resources, inadequate training for professional caregivers, and lack of support for informal carers (Alzheimer's Disease International 2014; Binns and Low 2024; Cheng et al. 2013). Therefore, there is a need to critically review past and recent research in YOD within the Asia‐Pacific region to better understand the scope of research and identify research gaps that can be addressed to inform regionally relevant care and policy development.

This scoping review aimed to comprehensively map the existing literature on YOD broadly, rather than specific YOD subtypes, from the Asia‐Pacific region and provide a comprehensive overview of studies published to December 2024. Specifically, our questions were: (1) Which Asia‐Pacific countries/territories have published YOD research, and how has the volume of publications changed over time? (2) What are the YOD research topics in the Asia‐Pacific region? (3) What are the designs of studies focused on these research topics? Thus, it was not the aim of this review to summarize what is known about YOD research in the Asia‐Pacific region, but rather to first canvas the research landscape, providing valuable insights into the characteristics of YOD within Asia‐Pacific and identifying key gaps in the YOD literature. Our study could promote future YOD collaborative research with a regional focus and inform culturally and contextually appropriate interventions and policies to better address the needs of individuals with YOD and their families in the Asia‐Pacific region.

## 2 Methods

This scoping review followed the PRISMA (Preferred Reporting Items for Systematic reviews and Meta‐Analyzes) extension for scoping reviews (PRISMA‐ScR) guidelines (Tricco et al. 2018).

### 2.1 Eligibility Criteria

We included publications related to YOD in the Asia‐Pacific region. YOD was defined as dementia of any cause with symptom onset prior to age 65. A list of 42 Asia‐Pacific countries and territories is listed in Table 1 (United States Department of Transportation 2024). All empirical studies, including case reports/series, quantitative and qualitative research, and mixed‐methods research, were included. The term “carers” is used to refer to informal carers, that is unpaid, often family members, providing care.

**TABLE 1:** List of Asia‐Pacific countries and territories^a^.

Afghanistan	Indonesia	Nepal	Solomon Islands
Australia	Japan	New Caledonia	South Korea
Bangladesh	Kiribati	New Zealand	Sri Lanka
Bhutan	Laos	North Korea	Taiwan
Brunei	Malaysia	Niue	Thailand
Cambodia	Maldives	Pakistan	Timor‐Leste
China (+ Hong Kong and Macau)	Marshall Islands	Palau	Tonga
Cook Islands	Micronesia	Papua New Guinea	Tuvalu
Fiji	Myanmar	Philippines	Vanuatu
India	Mongolia	Singapore	Vietnam

^a^ From United States Department of Transportation, Federal Aviation Administration, areas of responsibility in Asia‐Pacific https://www.faa.gov/about/office_org/headquarters_offices/apl/international_affairs/asia_pacific/countries.

Inclusion criteria were (all must be met):

1. Studies involving human participants with YOD or informal carers of people with YOD.
2. Participants living in one of the 42 countries or territories in the Asia‐Pacific region (Table 1).
3. Studies were published in either English or Chinese, as one of the authors was fluent in Chinese and able to accurately screen, extract, and assess data from these publications.
4. Studies were published from any date until December 31, 2024.

Exclusion criteria were:

1. Studies conducted by researchers located in the Asia‐Pacific region but did not involve participants or data from Asia‐Pacific countries/territories.
2. Nonempirical studies, including systematic reviews, meta‐analyzes, and scoping reviews.
3. Nonpeer‐reviewed studies, including editorials, commentaries, book chapters, and conference abstracts.
4. Studies published in languages other than English or Chinese.

### 2.2 Search Strategies

To identify potentially relevant studies, we searched Scopus and PsycINFO from their inception date to December 31, 2024. Search terms were drafted and refined through discussion among the authors: (“young‐onset” AND “dementia”) OR (“young” AND “onset” AND “dementia”) OR (“younger‐onset” AND “dementia”) OR (“younger” AND “onset” AND “dementia”) OR (“early‐onset” AND “dementia”) OR (“early” AND “onset” AND “dementia”) OR (“presenile” AND “dementia”) OR (“juvenile” AND “dementia”). “Affiliation country” limits were used to restrict the search to studies published in the Asia‐Pacific region (see Table 1). We also scanned the reference lists of included studies to identify additional studies.

### 2.3 Selection of Sources of Evidence

The first author performed an initial screening of titles and abstracts. Studies that met the eligibility criteria, or where the title and abstract lacked sufficient information for exclusion, were subjected to full‐text retrieval and further screening based on the inclusion and exclusion criteria. Any queries in the selection process were resolved through consensus with at least one other author.

### 2.4 Data Charting Process

A data‐charting Microsoft Excel table was developed to extract data from selected publications. The first author charted the data and discussed the results with the remaining authors to continuously update the data‐charting table and resolve any discrepancies.

### 2.5 Data Extraction

We extracted data from each included study related to our scoping review questions. In particular, article characteristics (e.g., title, authors, country/territories, publication year), research topics and study design (case report/series, case control, cohort, cross‐sectional, qualitative, mixed‐methods, experimental, or diagnostic accuracy) were analyzed. Research topics were organized into seven categories: (1) Biological and neuroimaging investigations (Genetics, Neuroimaging, Cerebrospinal fluid, Electroencephalogram, Blood, Multimodal investigations), (2) Clinical features and course, (3) Epidemiology and outcomes (Etiology and risk factors, Incidence/prevalence, Mortality, Health economics, Combined epidemiological studies), (4) Neuropathology, (5) Psychosocial impacts (Carers and families, Employment, Driving), (6) Support, intervention and services, and (7) Diagnosis and assessment. Two authors categorized the studies, and any disputes within these were resolved with a third author.

## 3 Results

**FIGURE 1:**
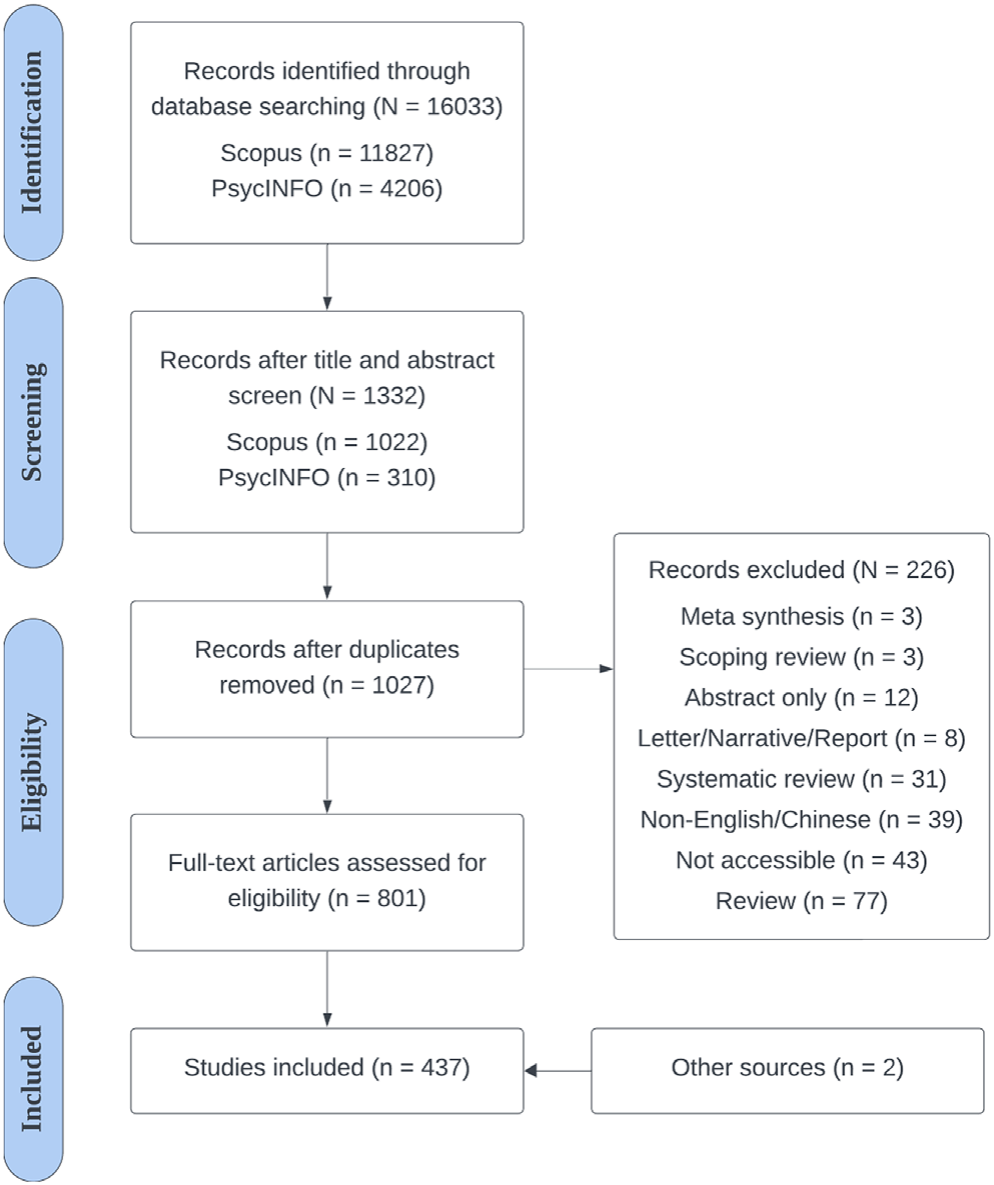
PRISMA‐ScR flowchart.

The PRISMA flow diagram in Figure 1 outlines the study selection process. The initial database search yielded 16 033 records. Following the screening of titles and abstracts, 1332 records remained. After removing duplicates, 1027 records were retained. An additional 226 records were excluded for various reasons. To minimize missing relevant literature, supplementary search methods were employed, including consultation with the subject experts, co‐authors. Full‐text screening of the remaining 801 articles and two articles from other sources, as described, resulted in the inclusion of 437 studies for data analysis and synthesis. These 437 studies are listed in Table S1.

### 3.1 Which Asia‐Pacific Countries/Territories Have Published YOD Research, and How Has the Volume of Publications Changed Over Time?

**FIGURE 2:**
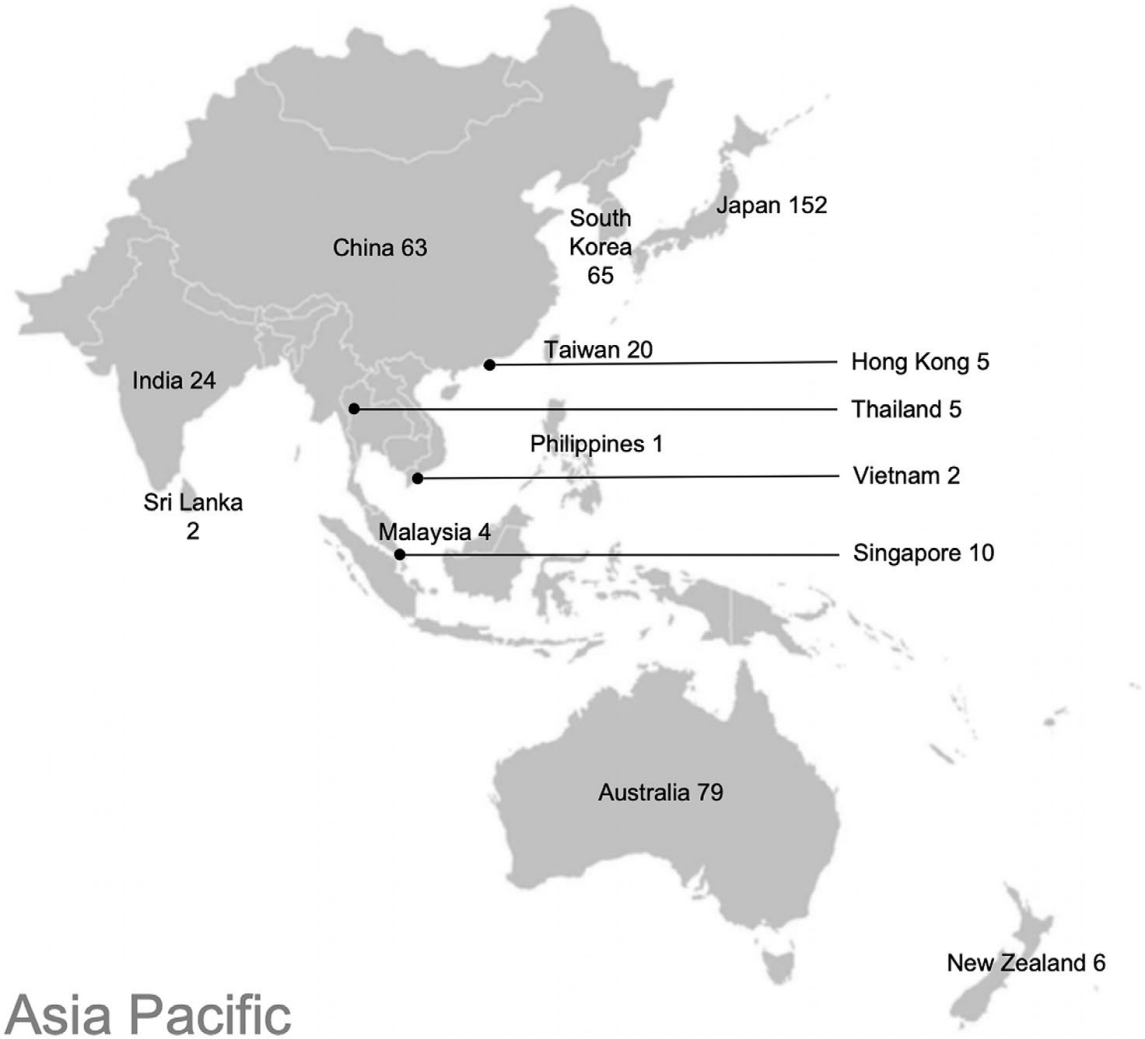
Number of studies by Asia‐Pacific country/territory.

**FIGURE 3:**
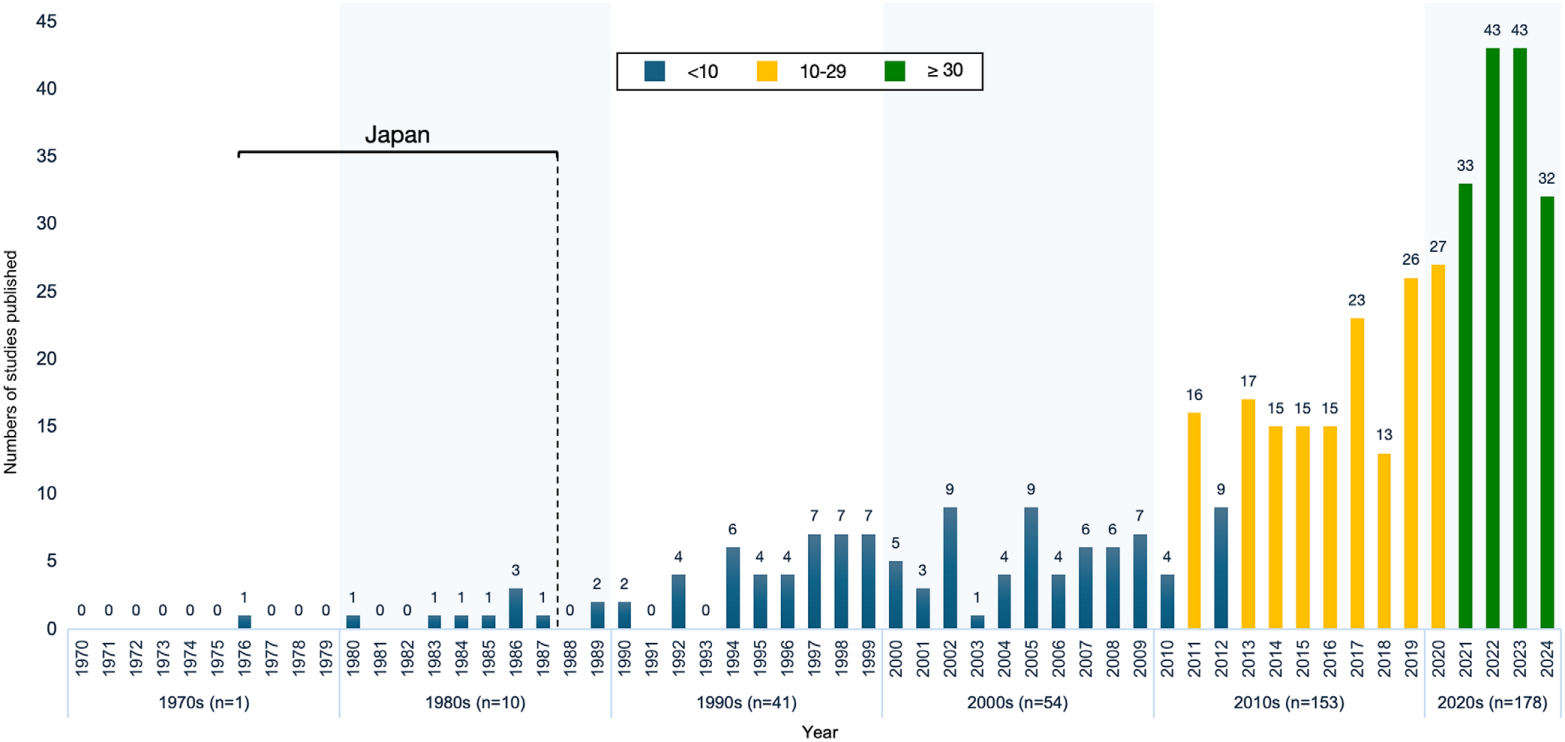
Timeline of publications in Asia‐Pacific (count per year).

Figure 2 shows the map of publications by country/territory. Among the 42 Asia–Pacific countries/territories, 14 have published YOD research. Japan has published the highest number of studies (*n* = 152, 34.7%), followed by Australia (*n* = 79; 18.1%), South Korea (*n* = 65; 14.9%), and China (*n* = 63; 14.4%) (Table S2). While most countries/territories began publishing YOD research in the 1990s (Figure 3), Japan published the earliest study in 1976. Figure 3 also shows the volume of YOD publications in the Asia‐Pacific region has increased steadily from the early 2010s.

### 3.2 What Are the YOD Research Topics in the Asia‐Pacific Region?

Table 2 shows the research topics of the included studies and Table S2 shows the research topics by country/territory. The largest category of studies focused on *Biological and neuroimaging investigations* (*n* = 189, 43.2%). Almost 30% of the included studies examined the genetic factors of YOD (*n* = 118; 27.0%); with the exception of Hong Kong, all 14 countries/territories with YOD research reported on genetics, including apolipoprotein E, presenilin, amyloid precursor protein, and other risk‐associated genes. These studies encompassed a wide spectrum of conditions, ranging from Alzheimer's disease to rarer causes such as Niemann–Pick disease and lupus. There were 43 studies (9.8%) investigating neuroimaging and YOD.

**TABLE 2:** Study design by research topics of the 437 empirical Asia‐Pacific studies.

Research topics	Case report/series	Case control	Cross‐sectional/Survey	Cohort	Qualitative	Mixed‐methods	Experimental	Diagnostic accuracy	Grand total
1. Biological and neuroimaging investigations	77	68	30	14					189
Genetics	73	22	19	4					118
Neuroimaging	3	27	9	4					43
Cerebrospinal fluid		10	1	2					13
Multimodal investigations	1	2	1	3					7
Electroencephalogram		5		1					6
Blood/Plasma		2							2
2. Clinical features and course	33	13	32	14	1				93
3. Epidemiology and outcomes	8	10	5	31					54
Etiology and risk factors	8	9	2	7					26
Incidence/prevalence				13					13
Mortality		1	2	8					11
Combined epidemiological studies				3					3
Health economics			1						1
4. Neuropathology	31	4	7	1					43
5. Psychosocial impacts		2	9		14	1			26
Carers and families			9		7	1			17
Employment		1			6				7
Driving		1			1				2
6. Support, intervention and services	3	1	8	1	5	3	3		24
7. Diagnosis and assessment			4	1	2			1	8
Grand total	152	98	95	62	22	4	3	1	437

The second largest category of studies focused on *Clinical features and course* (*n* = 93; 21.3%). With the exception of the Philippines and Vietnam, all 14 countries/territories with YOD research reported on *Clinical features and course*. Japan, South Korea and China have published 27 studies (17.8%), 10 studies (15.4%), and 12 studies (19.0%), respectively, on *Clinical features and course* of YOD. There were 54 studies (12.4%) in the category of *Epidemiology and outcomes*. Within this, 26 studies (6.0%) examined etiology and risk factors, and 13 studies (3.0%) investigated incidence/prevalence. The *Neuropathology* category accounted for 43 studies (9.8%), with most of these studies (87.5%) conducted in Japan. The category of *Psychosocial impacts* encompassed 6.0% (*n* = 26) of total studies and within this category, there were 17 studies (3.9%) on carers and families and seven studies (1.6%) focused on employment. Twenty‐four studies (5.5%) were in the category of *Support, intervention, and services*, while eight studies (1.8%) were in the *Diagnosis and assessment* category. Two‐thirds (66.7%) of the studies on Support, interventions and services came from Australia.

Japan, South Korea, and China have published mostly biological and neuropathology research (*n* = 101, 66.4%; *n* = 38, 58.5%; *n* = 49, 77.8%, respectively). In contrast, Australia has published more evenly across the seven categories of research topics, with 12 studies (15.2%) on *Biological and neuroimaging* research, 16 studies (20.3%) on *Clinical features and course*, 13 studies (16.5%) on *Epidemiology and outcomes*, 12 studies (15.2%) on *Psychosocial impacts*, and 16 studies (20.3%) on *Support, interventions*, *and services*.

**FIGURE 4:**
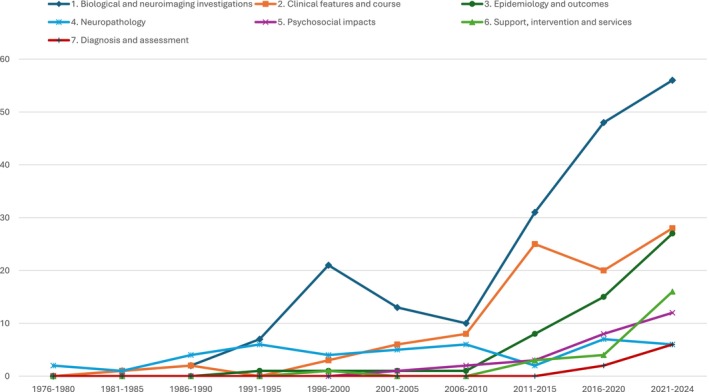
Number of publications of research topics over time.

With regards to the trends of publications of research topics over time (Figure 4), the category of *Biological and neuroimaging investigations* continues to increase. There has been a general increase in the number of studies published in the categories of *Epidemiology and outcomes*, *Psychosocial impacts*, and *Supports, interventions and services* since the mid‐2000s. In contrast, publications on *Neuropathology* in YOD have plateaued since the early 2000s.

### 3.3 What Are the Study Designs of These Research Topics?

Among the 437 empirical studies, over one‐third were case reports or case series (*n* = 152; 34.8%) (Table 2). Other common study designs included case–control (*n* = 98; 22.4%), cohort (*n* = 62; 14.2%), and cross‐sectional (*n* = 95; 21.7%) studies. The remaining 11% consisted of qualitative studies (*n* = 22; 5.0%), mixed‐methods studies (*n* = 4; 0.9%), experimental studies (*n* = 3; 0.7%), and diagnostic accuracy (*n* = 1; 0.2%). Table S3 shows study type published by each country/territory.

## 4 Discussion

This scoping review aimed to understand the current research foci in YOD in the Asia‐Pacific region, by examining the countries/territories that have published YOD research, their research topics and methodology. There has been an increasing number of studies published in YOD over the decades, from < 10 per year prior to 2010 to > 30 per year since 2021. Four countries, Japan, Australia, South Korea, and China, account for 82.2% of all YOD studies identified in this review. YOD research topics have predominantly been in the domains of *Biological and neuroimaging investigations* (43.2%) and *Clinical features and course* (21.3%). Qualitative research remains limited in the YOD literature, despite its potential to enhance cultural understanding of the lived experiences of people with YOD and their families. Although only 5.0% of YOD studies identified in this review employed qualitative methods overall, such approaches were used in 53.8% of studies examining psychosocial impacts on carers and families, employment, and driving, indicating the value of further qualitative research in these domains. Co‐design or co‐creation of interventions and support services can help ensure they are culturally appropriate, relevant, and fit for purpose.

Genetics represents a significant focus with the highest number of studies (27.0%), with the majority (81.4%) of genetics studies coming from Japan, China, and South Korea. Worldwide, current genetic knowledge and relationships with outcomes, such as mortality, in different types of YOD have been generated by cohorts of European ancestry (Moore et al. 2020). Importantly though, due to inconsistencies seen in the pathogenic genes implicated in frontotemporal dementia (FTD), where in Korean FTD populations, microtubule‐associated protein tau (*MAPT*), progranulin (*GRN*), and chromosome 9 open reading frame 72 (*C9orf72*) genes are rare (Kim et al. 2022), with instead other genes being implicated (Kim et al. 2018), whereas *C9orf72* was the most common pathogenic cause of FTD in the Singapore and Philippines population (Tan et al. 2023). This emphasizes the importance of continuing to conduct genetic studies across the Asia‐Pacific region.

Important gaps in the research include lack of studies in the categories of *Psychosocial impacts* (5.9%), *Support, interventions and services* (5.5%), and *Diagnosis and assessment* (1.8%). This is significant because diagnosing YOD can be challenging due to the overlap with psychiatric disorders and atypical presentations. Furthermore, the diverse contexts within which symptoms manifest contribute to difficulties in accurate identification. Within the studies that had been conducted, delays in diagnosis ranged from 2 years to 3.5 years (Ellajosyula et al. 2022; Loi et al. 2022; Sharma et al. 2024), similar to durations reported in European studies (van Vliet et al. 2013). The use of fluid and imaging biomarkers in the assessment of YOD provides valuable diagnostic information and can aid in differentiating primary psychiatric disorders from dementia (Chouliaras and O'Brien 2023; Loi et al. 2025, 29; Vipin et al. 2022), and there is a need to replicate these results with more diverse cultural populations. The psychosocial impacts of having dementia at a younger age were largely explored in Australia and Japan, with most studies focusing on carers and families. Different cultural expectations of family and carers and stigma and shame can impact carers as well as affect recognition of symptoms and need for support (Franzen et al. 2023). While there were studies on family carers, there were scarce studies on the psychosocial impacts of children who have a parent with YOD and the psychosocial impacts of people with YOD.

Most studies examining support, interventions and services were from Australia. These mostly pertained to service usage and support, with only one study reporting on an intervention, a combined music therapy and cognitive behavior therapy program for couples affected by YOD (Loi et al. 2024). More studies are needed to investigate support services that are culturally and socially appropriate, including the use of respite, day programs and residential care, in the Asia‐Pacific region considering there may be different health structures and accessibility. Furthermore, as Alzheimer's disease in particular moves into an era that includes disease modifying treatments, it will be important for research that investigates the effects of these current and emerging interventions to include younger people with dementia from across the Asia‐Pacific region. There were few studies investigating employment, finances, driving and the financial impacts of YOD in the Asia‐Pacific region. As the prevalence of YOD continues to increase, this will place a greater burden on Asia‐Pacific countries than before. Hence, there is a growing need for research focused on diagnosis, assessment, epidemiology, and support services. Such research is essential to inform the development and implementation of region‐specific interventions aimed at prevention, early detection, and effective care within countries and regions with unique multicultural and multiethnic societies.

We found that only a third of the 42 Asia‐Pacific countries/territories have published research in YOD. Of the Pacific countries in this region, there have only been studies from Australia and New Zealand. We did not identify any YOD studies from the Pacific islands. Many Pacific islands are remotely located and are considered developing nations, based on economic measures with subsequent poor infrastructure and less developed healthcare systems. These sociodemographic characteristics, in addition to lifestyle factors, are potentially risk factors for dementia, and their respective Indigenous populations may be at higher risk of developing dementia at a younger age (Clarke et al. 2024). While New Zealand is considered a high‐income country, Māori, the Indigenous people of New Zealand, as well as Pacific peoples living in New Zealand, experience a higher burden of dementia risk factors (Ma'u et al. 2021), present with dementia at a younger age (Cullum et al. 2018), and have a higher prevalence of YOD (Ryan et al. 2022). While Australia has published almost 20% of included empirical studies, most of these studies included predominately Caucasian participants, with very few Indigenous Australians and culturally and linguistically diverse people included—thus there is still limited knowledge about dementia in Indigenous Australians, who, similar to Māori and Pacific peoples, also present with dementia at a younger age (Smith et al. 2008). Policy makers and researchers across the Asia‐Pacific region could play a key role in supporting and building research capacity in underrepresented countries and populations, enabling the generation of much‐needed knowledge on YOD.

### 4.1 Strength and Limitations

The main strength of this review is identifying the scope of research conducted in Asia‐Pacific, including where and what type of research is being done in each country/territory, thus contributing to knowledge about YOD in particular regions. Gaps have been identified, including the lack of contribution from various Asia‐Pacific countries/territories and areas of research which are needed for the future.

We need to acknowledge a few limitations. First, the review focused broadly on YOD, with search terms of “young‐onset” and “early‐onset,” so studies which investigated specific subtypes such as frontotemporal dementia or Alzheimer's disease variants such as posterior cortical atrophy may have been missed. Following on from this, the search strategy may also have led to a bias toward studies which investigated “young‐onset” or “early‐onset” Alzheimer's disease. Hereditary forms of YOD, including like Huntington's disease, were not specifically included in the search terms. We also focused on the search term “dementia” and did not include “neurocognitive disorder” because “young‐onset neurocognitive disorder” is not a commonly used term. Thesaurus (controlled vocabulary) terms, including the APA Thesaurus, were not used in this search strategy. Instead, we used a combination of free‐text keywords and synonyms to capture relevant literature across Scopus and PsycINFO. Second, while the methodologies of included studies were described, no formal quality assessment was conducted for this scoping review. Databases such as PubMed or CINAHL were not used which might have limited the articles we found. Scopus was chosen because it offers extensive multidisciplinary indexing, including health, medical and social science journals and it provides 100% coverage of MEDLINE, EMBASE and Compendex (Burnham 2006). Similarly, articles published in languages other than Chinese or English may have been missed. We acknowledge that only a single reviewer was used for initial screening. We also did not perform an in‐depth review of each included study across the seven research topic categories. Future work is planned to analyze each of the research topic categories and subcategories (e.g., genetics, cerebrospinal fluid, electroencephalogram, mortality, carers and families, employment) to better understand the research landscape of these unique topics within the Asia‐Pacific region. Third, there is no universally accepted definition of the Asia‐Pacific region. We chose the U.S. Federal Aviation Administration's list of Asia‐Pacific countries and territories, as it encompasses more areas (*n* = 42) than other commonly used definitions. For example, the Alzheimer's Disease International has 19 member associations in the Asia Pacific region; the two World Health Organization Regions has 38 countries in Asia‐Pacific (OCED 2024). Fourth, approximately 30% of the empirical studies were case reports or case series, highlighting a potential publication bias that should be considered when interpreting the findings. Lastly, YOD research is a rapidly evolving field. We included studies published up to December 2024. We plan to repeat this review in 5 years to capture further developments in YOD research in the Asia‐Pacific region.

## 5 Conclusion

This scoping review identified 437 YOD research articles published in the Asia‐Pacific region until the end of 2024. Only a third of the Asia‐Pacific countries have published YOD research, with Japan, Australia, South Korea, and China contributing to 82.2% of all YOD research in this region. The volume of YOD publications in the Asia‐Pacific region has increased steadily from the early 2010s, with the focus of the YOD research landscape dominated by biological research. There is an urgent need for research in all Asia–Pacific countries/territories to better understand the journey of YOD and to co‐develop interventions and services that address the needs of those affected. Collaboration between the Asia–Pacific YOD research communities, people with lived experience, and health and social service policy makers is essential to ensure that research is targeted, relevant, and leads to improved access to medical and psychosocial treatment and support based on the best available local evidence.

## Funding

This work was supported by the National Health and Medical Research Council and the Auckland School of Medicine.

## Conflicts of Interest

The authors declare no conflicts of interest.

## Supporting information


**Table S1:** List of the 437 empirical studies included in this scoping review.
**Table S2:** Research topics of the 437 empirical Asia‐Pacific studies by countries/territories.
**Table S3:** Study type published by each Asia‐Pacific country/territory.

## Data Availability

The data that support the findings of this study are available from the corresponding author upon reasonable request.
