# Supplementary material for: Mapping the Young‐Onset Dementia Research in the Asia‐Pacific Region: A Scoping Review
Source: Asia Pac Psychiatry. 2026 Jul 14;18(3):e70030. doi: 10.1111/appy.70030 (PMC13369418; doi:10.1111/appy.70030)
Supplement: Supplementary file 1 — Table S1: List of the 437 empirical studies included in this scoping review. Table S2: Research topics of the 437 empirical Asia‐Pacific studies by countries/territories. Table S3: Study type published by each Asia‐Pacific country/territory. [file APPY-18-e70030-s001.docx]

**Supplementary table S1:** List of the 437 empirical studies included in this scoping review

| **Category** | **Topic** | **Year** | **Country** | **Authors** | **Title** | **Journal** | **Type** |
| --- | --- | --- | --- | --- | --- | --- | --- |
| 1. Biological and neuroimaging investigations | Blood/Plasma | 2015 | South Korea | Kim et al. | Elevation of the plasma Abeta40/Abeta42 ratio as a diagnostic marker of sporadic early-onset Alzheimer's disease | Journal of Alzheimer’s Disease, 48(4), 1043–1050. | Case control |
| 1. Biological and neuroimaging investigations | Blood/Plasma | 2024 | Taiwan | Chang et al. | Clinical significance of the plasma biomarker panels in amyloid-negative and tau PET-positive amnestic patients: Comparisons with Alzheimer’s disease and unimpaired cognitive controls | International Journal of Molecular Sciences, 25(11), 5607. | Case control |
| 1. Biological and neuroimaging investigations | Cerebrospinal fluid | 1986 | Japan | Nakano et al. | Acetylcholinesterase activity in cerebrospinal fluid of patients with Alzheimer's disease and senile dementia | J Neurol Sci, 75(2), 213–223. | Case control |
| 1. Biological and neuroimaging investigations | Cerebrospinal fluid | 1990 | Japan | Kawakatsu et al. | Acetylcholinesterase activities and monoamine metabolite levels in the cerebrospinal fluid of patients with Alzheimer's disease | Biological Psychiatry (1969), 28(5), 387–400. | Case control |
| 1. Biological and neuroimaging investigations | Cerebrospinal fluid | 1995 | Japan | Yamada et al. | Decreased interleukin-6 level in the cerebrospinal fluid of patients with Alzheimer-type dementia | Neuroscience Letters, 186(2), 219–221. | Case control |
| 1. Biological and neuroimaging investigations | Cerebrospinal fluid | 1997 | Japan | Song et al. | Cerebrospinal fluid apo E and apo A-I concentrations in early- and late- onset Alzheimer's disease | Neuroscience Letters, 231(3), 175–178. | Case control |
| 1. Biological and neuroimaging investigations | Cerebrospinal fluid | 1998 | Japan | Tsugu et al. | High levels of hippocampal cholinergic neurostimulating peptide (HCNP) in the CSF of some patients with Alzheimer's disease | European Journal of Neurology, 5(6), 561. | Case control |
| 1. Biological and neuroimaging investigations | Cerebrospinal fluid | 2002 | Japan | Matsuda et al. | Measurement of laminins in the cerebrospinal fluid obtained from patients with Alzheimer's disease and vascular dementia using a modified enzyme-linked immunosorbent assay | Dementia and Geriatric Cognitive Disorders, 14(3), 113–122. | Case control |
| 1. Biological and neuroimaging investigations | Cerebrospinal fluid | 2013 | Japan | Ikeda et al. | Cerebrospinal fluid levels of phosphorylated tau and Aβ1-38/Aβ1- 40/Aβ1-42 in Alzheimer's disease with PS1 mutations | Amyloid, 20(2), 107–112. | Case control |
| 1. Biological and neuroimaging investigations | Cerebrospinal fluid | 2020 | Singapore | Yatawara et al. | Small vessel disease and associations with cerebrospinal fluid amyloid, tau, and neurodegeneration (ATN) biomarkers and cognition in young onset dementia | Journal of Alzheimer’s Disease, 77(3), 1305–1314. | Case control |
| 1. Biological and neuroimaging investigations | Cerebrospinal fluid | 2021 | Singapore | Tan et al. | Altered cerebrospinal fluid exosomal microRNA levels in young-onset Alzheimer's disease and frontotemporal dementia | JAD Reports, 5(1), 805–813. | Case control |
| 1. Biological and neuroimaging investigations | Cerebrospinal fluid | 2022 | Australia | Walia et al. | Cerebrospinal fluid neurofilament light predicts the rate of executive function decline in younger-onset dementia | Journal of the Neurological Sciences, 432, Article 120088. | Cohort |
| 1. Biological and neuroimaging investigations | Cerebrospinal fluid | 2022 | Singapore | Soo et al. | Safety and usefulness of lumbar puncture for the diagnosis and management of young-onset cognitive disorders | Journal of Alzheimer’s Disease, 87(1), 479–488. | Cohort |
| 1. Biological and neuroimaging investigations | Cerebrospinal fluid | 2023 | Australia | Walia et al. | Cerebrospinal fluid neurofilament light and cerebral atrophy in younger-onset dementia and primary psychiatric disorders | Internal Medicine Journal, 53(9), 1564–1569. | Cross-sectional/Survey |
| 1. Biological and neuroimaging investigations | Cerebrospinal fluid | 2023 | China | Lei et al. | CSF biomarkers for early-onset Alzheimer's disease in Chinese population from PUMCH dementia cohort | Frontiers in Neurology, 13, 1030019. | Case control |
| 1. Biological and neuroimaging investigations | Electroencephalogram | 1994 | Japan | Miyauchi et al. | Quantitative EEG in patients with presenile and senile dementia of the Alzheimer type | Acta Neurologica Scandinavica, 89(1), 56–64. | Case control |
| 1. Biological and neuroimaging investigations | Electroencephalogram | 1997 | Japan | Wada et al. | Electroencephalographic abnormalities in patients with presenile dementia of the Alzheimer type: Quantitative analysis at rest and during photic stimulation | Biological Psychiatry (1969), 41(2), 217–225. | Case control |
| 1. Biological and neuroimaging investigations | Electroencephalogram | 1998 | Japan | Wada et al. | Abnormal functional connectivity in Alzheimer's disease: Intrahemispheric EEG coherence during rest and photic stimulation | European Archives of Psychiatry and Clinical Neuroscience, 248(4), 203–208. | Case control |
| 1. Biological and neuroimaging investigations | Electroencephalogram | 1998 | Japan | Wada et al. | Reduced interhemispheric EEG coherence in Alzheimer disease: Analysis during rest and photic stimulation | Alzheimer Disease and Associated Disorders, 12(3), 175–181. | Case control |
| 1. Biological and neuroimaging investigations | Electroencephalogram | 2021 | China | Lin et al. | Differences in multimodal electroencephalogram and clinical correlations between early-onset Alzheimer’s disease and frontotemporal dementia | Frontiers in Neuroscience, 15, 687053. | Case control |
| 1. Biological and neuroimaging investigations | Electroencephalogram | 2023 | Australia | Brown et al. | Electroencephalography in young onset dementia | BMC Neurology, 23(1), Article 202. | Cohort |
| 1. Biological and neuroimaging investigations | Genetics | 1995 | Australia | Martins et al. | ApoE genotypes in australia: Roles in early and late onset alzheimer’s disease and down’s syndrome | Neuroreport, 6(11), 1513–1516. | Case control |
| 1. Biological and neuroimaging investigations | Genetics | 1995 | Japan | Kamino et al. | Linkage and haplotype analysis of familial early-onset Alzheimer disease in Japanese population | The Japanese journal of human genetics, 40(3), 229–241. | Case control |
| 1. Biological and neuroimaging investigations | Genetics | 1996 | Japan | Nunomura et al. | Apolipoprotein E polymorphism and susceptibility to early- and late-onset sporadic Alzheimer's disease in Hokkaido, the northern part of Japan | Neuroscience Letters, 206(1), 17–20. | Case control |
| 1. Biological and neuroimaging investigations | Genetics | 1996 | Japan | Matsumura et al. | Japanese siblings with missense mutation (717Val->Ile) in amyloid precursor protein of early-onset Alzheimer's disease | Neurology, 46(6), 1721–1723. | Case report/series |
| 1. Biological and neuroimaging investigations | Genetics | 1996 | Japan | Isoe et al. | Presenilin-1 polymorphism in patients with Alzheimer's disease, vascular dementia and alcohol-associated dementia in Japanese population | Acta Neurologica Scandinavica, 94(5), 326–328. | Case control |
| 1. Biological and neuroimaging investigations | Genetics | 1996 | Japan | Tanahashi et al. | Sequence analysis of presenilin-1 gene mutation in Japanese Alzheimer’s disease patients. | Neuroscience Letters, 218(2), 139–141. | Cross-sectional/Survey |
| 1. Biological and neuroimaging investigations | Genetics | 1997 | Australia | Dark | A family with autosomal dominant, non-Alzheimer's presenile dementia | The Australian and New Zealand journal of psychiatry, 31(1), 139–144. | Case report/series |
| 1. Biological and neuroimaging investigations | Genetics | 1997 | Australia | Kwok et al. | Two novel (M233T and R278T) presenilin-1 mutations in early-onset Alzheimer's disease pedigrees and preliminary evidence for association of presenilin-1 mutations with a novel phenotype | Neuroreport, 8(6), 1537–1542. | Cohort |
| 1. Biological and neuroimaging investigations | Genetics | 1997 | Japan | Utatsu et al. | Autosomal dominant early onset dementia and leukoencephalopathy in a Japanese family: Clinical, neuroimaging and genetic studies | Journal of the Neurological Sciences, 147(1), 55–62. | Case report/series |
| 1. Biological and neuroimaging investigations | Genetics | 1998 | Japan | Kamimura et al. | Familial Alzheimer's disease genes in Japanese | Journal of the Neurological Sciences, 160(1), 76–81. | Cross-sectional/Survey |
| 1. Biological and neuroimaging investigations | Genetics | 1999 | Japan | Nakayama et al. | Apolipoprotein E phenotypes in healthy normal controls and demented subjects with Alzheimer's disease and vascular dementia in Mie prefecture of Japan | Psychiatry and Clinical Neurosciences, 53(6), 643–648. | Case control |
| 1. Biological and neuroimaging investigations | Genetics | 1999 | Japan | Iijima et al. | A distinct familial presenile dementia with a novel missense mutation in the tau gene | Neuroreport, 10(3), 497–501. | Cross-sectional/Survey |
| 1. Biological and neuroimaging investigations | Genetics | 1999 | Japan | Sugiyama et al. | A novel missense mutation (G209R) in exon 8 of the presenilin 1 gene in a Japanese family with presenile familial Alzheimer's disease. Mutation in brief no. 254. Online. | Human Mutation, 14(1), 90. | Case report/series |
| 1. Biological and neuroimaging investigations | Genetics | 1999 | Japan | Yasuda et al. | A pedigree with a novel presenilin 1 mutation at a residue that is not conserved in presenilin 2 | Archives of Neurology (Chicago), 56(1), 65–69. | Case report/series |
| 1. Biological and neuroimaging investigations | Genetics | 1999 | Japan | Honda et al. | Novel intronic polymorphisms in the presenilin-2 gene and a case-control association study of Alzheimer's disease | Psychiatry and Clinical Neurosciences, 53(5), 579–585. | Case control |
| 1. Biological and neuroimaging investigations | Genetics | 2000 | Japan | Yasuda et al. | A Japanese patient with frontotemporal dementia and parkinsonism by a tau P301S mutation | Neurology, 55(8), 1224–1227. | Case report/series |
| 1. Biological and neuroimaging investigations | Genetics | 2001 | Japan | Miyamoto et al. | Familial frontotemporal dementia and parkinsonism with a novel mutation at an intron 10+11-splice site in the tau gene | Annals of Neurology, 50(1), 117–120. | Case report/series |
| 1. Biological and neuroimaging investigations | Genetics | 2002 | Australia | Taddei et al. | Association between presenilin-1 Glu318Gly mutation and familial Alzheimer's disease in the Australian population | Molecular Psychiatry, 7(7), 776–781. | Case control |
| 1. Biological and neuroimaging investigations | Genetics | 2002 | Japan | Kondo et al. | Heterogeneity of presenile dementia with bone cysts (Nasu-Hakola disease): Three genetic forms | Neurology, 59(7), 1105–1107. | Cross-sectional/Survey |
| 1. Biological and neuroimaging investigations | Genetics | 2002 | Japan | Matsubara-Tsutsui et al. | Molecular evidence of presenilin 1 mutation in familial early onset dementia | American Journal of Medical Genetics, 114(3), 292–298. | Case report/series |
| 1. Biological and neuroimaging investigations | Genetics | 2002 | Japan | Saito et al. | Early-onset, rapidly progressive familial tauopathy with R406W mutation | Neurology, 58(5), 811–813. | Case report/series |
| 1. Biological and neuroimaging investigations | Genetics | 2002 | Japan | Takao et al. | A novel mutation (G217D) in the Presenilin 1 gene (PSEN1) in a Japanese family: Presenile dementia and parkinsonism are associated with cotton wool plaques in the cortex and striatum | Acta Neuropathologica, 104(2), 155–170. | Case report/series |
| 1. Biological and neuroimaging investigations | Genetics | 2002 | Japan | Hirono et al. | The effect of APOE epsilon4 allele on cerebral glucose metabolism in AD is a function of age at onset | Neurology, 58(5), 743. | Cross-sectional/Survey |
| 1. Biological and neuroimaging investigations | Genetics | 2004 | Japan | Tanahashi et al. | Association between Tau polymorphism and male early-onset Alzheimer's disease | NeuroReport, 15(1), 175–179. | Case control |
| 1. Biological and neuroimaging investigations | Genetics | 2004 | Japan | Hattori et al. | A novel presenilin 1 mutation (Y154N) in a patient with early onset Alzheimer's disease with spastic paraparesis | Neuroscience Letters, 368(3), 319–322. | Case report/series |
| 1. Biological and neuroimaging investigations | Genetics | 2005 | Japan | shikawa et al. | A mutant PSEN1 causes dementia with Lewy bodies and variant Alzheimer's disease | Annals of Neurology, 57(3), 429–434. | Case report/series |
| 1. Biological and neuroimaging investigations | Genetics | 2008 | Japan | Ikeuchi et al. | Mutational analysis in early-onset familial dementia in the Japanese population: The role of PSEN1 and MAPT R406W Mutations | Dementia and Geriatric Cognitive Disorders, 26(1), 43–49. | Case control |
| 1. Biological and neuroimaging investigations | Genetics | 2008 | South Korea | Park et al. | Identification of PSEN1 and APP gene mutations in Korean patients with early-onset Alzheimer’s disease | Journal of Korean Medical Science, 23(2), 213–217. | Cross-sectional/Survey |
| 1. Biological and neuroimaging investigations | Genetics | 2009 | Japan | Kasuga et al. | Depression and psychiatric symptoms preceding onset of dementia in a family with early-onset Alzheimer disease with a novel PSEN1 mutation | Journal of Neurology, 256(8), 1351–1353. | Case report/series |
| 1. Biological and neuroimaging investigations | Genetics | 2009 | Sri Lanka + India | De et al. | Cerebral autosomal dominant arteriopathy with subcortical infarcts and leukoencephalopathy (CADASIL): A patient from Sri Lanka | Journal of Clinical Neuroscience, 16, 1492–1493. | Case report/series |
| 1. Biological and neuroimaging investigations | Genetics | 2011 | India | Narayanaswamy et al. | Is there a familial overlap between dementia and other psychiatric disorders? | International Psychogeriatrics, 23(5), 749–755. | Case control |
| 1. Biological and neuroimaging investigations | Genetics | 2011 | Japan | Kobayashi et al. | Apolipoprotein E4 frequencies in a Japanese population with Alzheimer's disease and dementia with Lewy bodies | PloS One, 6(4), e18569. | Case control |
| 1. Biological and neuroimaging investigations | Genetics | 2011 | Japan | Numasawa et al. | Nasu-Hakola disease with a splicing mutation of TREM2 in a Japanese family | European Journal of Neurology, 18(9), 1179–1183. | Case report/series |
| 1. Biological and neuroimaging investigations | Genetics | 2011 | Japan | Shimada et al. | Clinical course of patients with familial early-onset Alzheimer's disease potentially lacking senile plaques bearing the E693DELTA mutation in amyloid precursor protein | Dementia and Geriatric Cognitive Disorders, 32(1), 45–54. | Case report/series |
| 1. Biological and neuroimaging investigations | Genetics | 2012 | Australia | Dobson-Stone et al. | C9ORF72 repeat expansion in clinical and neuropathologic frontotemporal dementia cohorts | Neurology, 79(10), 995–1001. | Case control |
| 1. Biological and neuroimaging investigations | Genetics | 2012 | Japan | Abe et al. | Phenotypical difference of amyloid precursor protein (APP) V717L mutation in Japanese family | BMC Neurology, 12(1), Article 38. | Case report/series |
| 1. Biological and neuroimaging investigations | Genetics | 2012 | Japan | Ishizuka et al. | Different clinical phenotypes in siblings with a presenilin-1 P264L mutation | Dementia and Geriatric Cognitive Disorders, 33(2–3), 132–140. | Case report/series |
| 1. Biological and neuroimaging investigations | Genetics | 2012 | Japan | Kinoshita et al. | Hereditary diffuse leukoencephalopathy with axonal spheroids caused by R782H mutation in CSF1R: Case report | Journal of the Neurological Sciences, 318(1–2), 115–118. | Case report/series |
| 1. Biological and neuroimaging investigations | Genetics | 2013 | China | Ji et al. | Apolipoprotein E ε4 frequency is increased among chinese patients with frontotemporal dementia and Alzheimer's disease | Dementia and Geriatric Cognitive Disorders, 36(3–4), 163–170. | Case control |
| 1. Biological and neuroimaging investigations | Genetics | 2013 | China | Wang et al. | Correlation between Apolipoprotein E polymorphism and age at onset of Alzheimer's disease in a Chinese Han population | Zhong hua yi xue za zhi, 93(3), 182. | Cross-sectional/Survey |
| 1. Biological and neuroimaging investigations | Genetics | 2013 | Japan | Niwa et al. | Clinical and neuropathological findings in a patient with familial Alzheimer disease showing a mutation in the PSEN1 gene | Neuropathology, 33(2), 199–203. | Case report/series |
| 1. Biological and neuroimaging investigations | Genetics | 2014 | China | Niu et al. | A novel mutation in the PSEN2 gene (N141Y) associated with early-onset autosomal dominant Alzheimer's disease in a Chinese Han family | Neurobiology of Aging, 35(10), 2420.e1-2420.e5. | Case report/series |
| 1. Biological and neuroimaging investigations | Genetics | 2014 | China | Jiao et al. | Mutational analysis in early-onset familial Alzheimer's disease in Mainland China | Neurobiology of Aging, 35(8), 1957.e1-1957.e6. | Case control |
| 1. Biological and neuroimaging investigations | Genetics | 2014 | China | Peng et al. | Novel APP K724M mutation causes Chinese early-onset familial Alzheimer's disease and increases amyloid-β42 to amyloid-β40 ratio | Neurobiology of Aging, 35(11), 2657.e1-2657.e6. | Case control |
| 1. Biological and neuroimaging investigations | Genetics | 2014 | Japan | Yagi et al. | Detecting gene mutations in Japanese Alzheimer's patients by semiconductor sequencing | Neurobiology of Aging, 35(7), 1780.e1-1780.e5. | Case control |
| 1. Biological and neuroimaging investigations | Genetics | 2015 | Australia | Jarmolowicz et al. | The patterns of inheritance in early-onset dementia: Alzheimer's disease and frontotemporal dementia | American Journal of Alzheimer’s Disease and Other Dementias, 30(3), 299–306. | Cross-sectional/Survey |
| 1. Biological and neuroimaging investigations | Genetics | 2015 | China | Jiang et al. | Identification of PSEN1 mutations p.M233L and p.R352C in Han Chinese families with early-onset familial Alzheimer's disease | Neurobiology of Aging, 36(3), 1602.e3-1602.e6. | Case report/series |
| 1. Biological and neuroimaging investigations | Genetics | 2015 | China | Xia et al. | Probable novel PSEN2 Pro123Leu mutation in a Chinese Han family of Alzheimer's disease | Neurobiology of Aging, 36(12), 3334.e13-3334.e18. | Case report/series |
| 1. Biological and neuroimaging investigations | Genetics | 2015 | Japan | Kutoku et al. | A second pedigree with amyloid-less familial Alzheimer’s disease harboring an identical mutation in the amyloid precursor protein gene (E693delta) | Internal Medicine, 54(2), 205–208. | Case report/series |
| 1. Biological and neuroimaging investigations | Genetics | 2015 | Japan | Sasaki et al. | Variable expression of microglial DAP12 and TREM2 genes in Nasu-Hakola disease | Neurogenetics, 16(4), 265–276. | Cross-sectional/Survey |
| 1. Biological and neuroimaging investigations | Genetics | 2015 | Malaysia | Ch’ng et al. | Identification of two novel mutations, PSEN I E280K and PRNP G127S, in a Malaysian family | Neuropsychiatric Disease and Treatment, 11(default), 2315–2322. | Case report/series |
| 1. Biological and neuroimaging investigations | Genetics | 2016 | China | Shi et al. | Frontotemporal dementia-related gene mutations in clinical dementia patients from a Chinese population | Journal of Human Genetics, 61(12), 1003–1008. | Case report/series |
| 1. Biological and neuroimaging investigations | Genetics | 2016 | South Korea | An et al. | A genetic screen of the mutations in the Korean patients with early-onset Alzheimer’s disease | Clinical Interventions in Aging, 11, 1817–1822. | Cross-sectional/Survey |
| 1. Biological and neuroimaging investigations | Genetics | 2016 | South Korea | An et al. | Novel PSEN1 G209A mutation in early-onset Alzheimer dementia supported by structural prediction | BMC Neurology, 16(1), Article 71. | Case report/series |
| 1. Biological and neuroimaging investigations | Genetics | 2016 | South Korea | Bagyinszky et al. | PSEN1 L226F mutation in a patient with early-onset Alzheimer’s disease in Korea | BMC Neurology, 16(1), Article 71. | Case report/series |
| 1. Biological and neuroimaging investigations | Genetics | 2017 | China | Sun et al. | Rapidly progressive frontotemporal dementia associated with MAPT mutation G389R | Journal of Alzheimer’s Disease, 55(2), 777–785. | Case report/series |
| 1. Biological and neuroimaging investigations | Genetics | 2017 | Malaysia | Chee et al. | A case of TREM2 mutation presenting with features of progressive non-fluent aphasia and without bone involvement | Australian and New Zealand Journal of Psychiatry, 51(11), 1157–1158. | Case report/series |
| 1. Biological and neuroimaging investigations | Genetics | 2017 | Malaysia | Chee et al. | A case of early-onset familial Alzheimer’s disease with both APP and novel PSEN2 mutations presenting with non-amnestic features | Australian and New Zealand Journal of Psychiatry, 51(12), 1252–1253. | Case report/series |
| 1. Biological and neuroimaging investigations | Genetics | 2017 | South Korea | Park et al. | A case of possibly pathogenic PSEN2 R62C mutation in a patient with probable early-onset Alzheimer's dementia supported by structure prediction | Clinical Interventions in Aging, 12, 367–375. | Case report/series |
| 1. Biological and neuroimaging investigations | Genetics | 2017 | South Korea | Park et al. | Identification of a novel PSEN1 mutation (Leu232Pro) in a Korean patient with early-onset Alzheimer's disease and a family history of dementia | Neurobiology of Aging, 56, Article 212. | Case report/series |
| 1. Biological and neuroimaging investigations | Genetics | 2018 | China | Xu et al. | The whole exome sequencing clarifies the genotype- phenotype correlations in patients with early-onset dementia | Aging and Disease, 9(4), 696–705. | Cross-sectional/Survey |
| 1. Biological and neuroimaging investigations | Genetics | 2018 | South Korea | Bagyinszky et al. | PSEN1 p.Thr116ile variant in two korean families with young onset Alzheimer’s disease | International Journal of Molecular Sciences, 19(9), Article 2604. | Case report/series |
| 1. Biological and neuroimaging investigations | Genetics | 2018 | Thailand | Giau et al. | Identification of a novel mutation in APP gene in a Thai subject with early-onset Alzheimer's disease | Neuropsychiatric Disease and Treatment, 14, 3015–3023. | Case report/series |
| 1. Biological and neuroimaging investigations | Genetics | 2019 | China | Jiang et al. | Mutation screening in Chinese patients with familial Alzheimer's disease by whole-exome sequencing | Neurobiology of aging, 76, 215.e15–215.e21. | Case control |
| 1. Biological and neuroimaging investigations | Genetics | 2019 | China | Li et al. | Two novel mutations and a de novo mutation in PSEN1 in early-onset Alzheimer’s disease | Aging and disease, 10(4), 908–914. | Case report/series |
| 1. Biological and neuroimaging investigations | Genetics | 2019 | China | Liu et al. | Diagnostic approach of early-onset dementia with negative family history: Implications from two cases of early-onset Alzheimer's disease with de novo PSEN1 mutation | Journal of Alzheimer’s Disease, 68(2), 551–558. | Case report/series |
| 1. Biological and neuroimaging investigations | Genetics | 2019 | China | Ma et al. | Gene mutations in a Han Chinese Alzheimer's disease cohort. | Brain and behavior, 9(1), e01180. | Case report/series |
| 1. Biological and neuroimaging investigations | Genetics | 2019 | China | Ni et al. | ACE gene missense mutation in a case with early-onset, rapid progressing dementia | General Psychiatry, 32(5), Article e100028. | Case report/series |
| 1. Biological and neuroimaging investigations | Genetics | 2019 | China | Wang et al. | Mutation and association analyses of dementia-causal genes in Han Chinese patients with early-onset and familial Alzheimer's disease | Journal of Psychiatric Research, 113, 141–147. | Case control |
| 1. Biological and neuroimaging investigations | Genetics | 2019 | China | Wang et al. | Probable novel PSEN1 Gln222Leu mutation in a Chinese family with early-onset Alzheimer's disease | Current Alzheimer Research, 16(8), 764–769. | Case report/series |
| 1. Biological and neuroimaging investigations | Genetics | 2019 | Japan | Kawazoe et al. | Sporadic case of young-onset rapidly progressive dementia with a novel frameshift mutation in exon 3 of CSF1R | Neurology and Clinical Neuroscience, 7(2), 103–104. | Case report/series |
| 1. Biological and neuroimaging investigations | Genetics | 2019 | Japan | Taminato et al. | Sporadic progressive myoclonic epilepsy with early-onset dementia caused by a de novo mutation in PSEN1 | Neurology and Clinical Neuroscience, 7(5), 294–296. | Case report/series |
| 1. Biological and neuroimaging investigations | Genetics | 2019 | South Korea | Bagyinszky et al. | Novel amyloid precursor protein mutation, Val669Leu (“Seoul APP”), in a Korean patient with early-onset Alzheimer's disease | Neurobiology of Aging, 84, 236.e1-236.e7. | Case report/series |
| 1. Biological and neuroimaging investigations | Genetics | 2019 | South Korea | Bagyinszky et al. | Early-onset Alzheimer's disease patient with prion (PRNP) p.Val180Ile mutation | Neuropsychiatric Disease and Treatment, 15, 2003–2013. | Case report/series |
| 1. Biological and neuroimaging investigations | Genetics | 2019 | South Korea | Jo et al. | Dopa responsive parkinsonism in an early onset Alzheimer's disease patient with a presenilin 1 mutation (A434T) | Journal of Alzheimer’s Disease, 71(1), 7–13. | Case report/series |
| 1. Biological and neuroimaging investigations | Genetics | 2019 | South Korea | Shen et al. | Novel GRN mutations in Koreans with Alzheimer’s disease | Molecular & Cellular Toxicology, 15(3), 345–352. | Case report/series |
| 1. Biological and neuroimaging investigations | Genetics | 2019 | South Korea | Van et al. | APP, PSEN1, and PSEN2 mutations in asian patients with early-onset Alzheimer disease | International Journal of Molecular Sciences, 20(19), Article 4757. | Cross-sectional/Survey |
| 1. Biological and neuroimaging investigations | Genetics | 2019 | South Korea | Van et al. | A pathogenic PSEN1 Trp165Cys mutation associated with early-onset Alzheimer's disease | BMC Neurology, 19(1), Article 188. | Case report/series |
| 1. Biological and neuroimaging investigations | Genetics | 2019 | Thailand | Van et al. | Analysis of 50 neurodegenerative genes in clinically diagnosed early-onset Alzheimer’s disease | International Journal of Molecular Sciences, 20(6), Article 1514. | Cross-sectional/Survey |
| 1. Biological and neuroimaging investigations | Genetics | 2020 | China | Zhang et al. | Association of rare variants in neurodegenerative genes with familial Alzheimer’s disease | Annals of Clinical and Translational Neurology, 7(10), 1985–1995. | Cross-sectional/Survey |
| 1. Biological and neuroimaging investigations | Genetics | 2020 | China | Han et al. | Genetic analysis of Chinese patients with early-onset dementia using next-generation sequencing | Clinical Interventions in Aging, 15, 1831–1839. | Case report/series |
| 1. Biological and neuroimaging investigations | Genetics | 2020 | China | Li et al. | A novel homozygous mutation in TREM2 found in a Chinese early-onset dementia family with mild bone involvement | tia family with mild bone involvement. Neurobiology of Aging, 86, 201.e1-201.e7. | Case report/series |
| 1. Biological and neuroimaging investigations | Genetics | 2020 | China | Zhou et al. | Presenilin 1 and app gene mutations in early-onset ad families from a southeast region of china | Current Alzheimer Research, 17(6), 540–546. | Case report/series |
| 1. Biological and neuroimaging investigations | Genetics | 2020 | South Korea | Bagyinszky et al. | Pathogenic PSEN1 Thr119Ile mutation in two Korean patients with early-onset Alzheimer's disease | Diagnostics (Basel), 10(6), Article 405. | Case report/series |
| 1. Biological and neuroimaging investigations | Genetics | 2020 | South Korea | Park et al. | Analysis of dementia-related gene variants in APOE ε4 noncarrying Korean patients with early-onset Alzheimer's disease | Neurobiology of Aging, 85, 155.e5-155.e8. | Case report/series |
| 1. Biological and neuroimaging investigations | Genetics | 2020 | South Korea | Vo et al. | Identification of a pathogenic PSEN1 Ala285Val mutation associated with early-onset Alzheimer's disease | Current Alzheimer Research, 17(5), 438–445. | Case report/series |
| 1. Biological and neuroimaging investigations | Genetics | 2020 | Thailand | Senanarong et al. | Pathogenic PSEN1 Glu184Gly mutation in a family from Thailand with probable autosomal dominant early onset Alzheimer’s disease | Diagnostics (Basel), 10(3), Article 135. | Case report/series |
| 1. Biological and neuroimaging investigations | Genetics | 2021 | China | Dong et al. | Effects of ApoE genotype on clinical phenotypes in early-onset and late-onset Alzheimer's disease in China: Data from the PUMCH dementia cohort | Brain and Behavior, 11(11), Article e2373. | Cross-sectional/Survey |
| 1. Biological and neuroimaging investigations | Genetics | 2021 | China | Jiao et al. | The role of genetics in neurodegenerative dementia: a large cohort study in South China | Npj Genomic Medicine, 6(1), Article 69. | Cross-sectional/Survey |
| 1. Biological and neuroimaging investigations | Genetics | 2021 | China | Mao et al. | Clinical phenotype and mutation spectrum of Alzheimer’s disease with causative genetic mutation in a chinese cohort | Current Alzheimer Research, 18(3), 265–272. | Case report/series |
| 1. Biological and neuroimaging investigations | Genetics | 2021 | China | Liu et al. | DHCR7 rs12785878 T>C polymorphism is associated with an increased risk of early onset of Alzheimer's disease in Chinese population | Frontiers in Genetics, 12, Article 583695. | Case control |
| 1. Biological and neuroimaging investigations | Genetics | 2021 | China | Li et al. | Novel PSEN1 and PSEN2 mutations identified in sporadic early-onset Alzheimer disease and posterior cortical atrophy | Alzheimer disease and associated disorders, 35(3), 208–213. | Case control |
| 1. Biological and neuroimaging investigations | Genetics | 2021 | Japan | Oka et al. | Japanese familial cases of frontotemporal dementia and parkinsonism with N279K tau gene mutation | Movement Disorders Clinical Practice (Hoboken, N.J.), 8(1), 126–132. | Case report/series |
| 1. Biological and neuroimaging investigations | Genetics | 2021 | Philippines | Dominguez et al. | Novel optineurin frameshift insertion in a family with frontotemporal dementia and parkinsonism without amyotrophic lateral sclerosis | Frontiers in Neurology, 12, 645913. | Case report/series |
| 1. Biological and neuroimaging investigations | Genetics | 2021 | South Korea | Bagyinszky et al. | A pathogenic presenilin-1 Val96Phe mutation from a Malaysian family | Alzheimer’s & Dementia, 15(7), P618–P618. | Case report/series |
| 1. Biological and neuroimaging investigations | Genetics | 2021 | Taiwan | Hsu et al. | Genetic study of young-onset dementia using targeted gene panel sequencing in Taiwan | American Journal of Medical Genetics. Part B, Neuropsychiatric Genetics, 186(2), 67–76. | Case control |
| 1. Biological and neuroimaging investigations | Genetics | 2022 | Australia | Huq et al. | Clinical impact of whole-genome sequencing in patients with early-onset dementia | Journal of Neurology, Neurosurgery and Psychiatry, 93(11), 1181–1189. | Cross-sectional/Survey |
| 1. Biological and neuroimaging investigations | Genetics | 2022 | China | Chen et al. | Very early-onset Alzheimer's disease in the third decade of life with de novo PSEN1 mutations | Journal of Alzheimer’s Disease, 85(1), 65–71. | Case report/series |
| 1. Biological and neuroimaging investigations | Genetics | 2022 | China | Li et al. | The identification of PSEN1 p.Tyr159Ser mutation in a non-canonic early-onset Alzheimer's disease family | Molecular and Cellular Neuroscience, 120, Article 103715. | Case report/series |
| 1. Biological and neuroimaging investigations | Genetics | 2022 | China | Li et al. | Case analysis of early-onset Alzheimer's disease associated with TBK1 p.Tyr235Phe gene mutation | Frontiers in Neurology, 13, Article 993399. | Case report/series |
| 1. Biological and neuroimaging investigations | Genetics | 2022 | China | Dong et al. | PSEN2 mutation spectrum and novel functionally validated mutations in Alzheimer's disease: Data from PUMCH dementia cohort | Journal of Alzheimer’s Disease, 87(4), 1549–1556. | Cohort |
| 1. Biological and neuroimaging investigations | Genetics | 2022 | India | Tallapalli et al. | A rare genetic cause of young onset rapidly progressive dementia- first report from India | Neurology India, 70(2), 781–783. | Case report/series |
| 1. Biological and neuroimaging investigations | Genetics | 2022 | South Korea | Shim et al. | A possible pathogenic PSEN2 Gly56Ser mutation in a Korean patient with early-onset Alzheimer’s disease | International Journal of Molecular Sciences, 23(6), Article 2967. | Case report/series |
| 1. Biological and neuroimaging investigations | Genetics | 2022 | South Korea | Shim et al. | Identification of the third case of PSEN1 Tyr389His variant in early-onset Alzheimer’s disease in Korea | International Journal of Molecular Sciences, 23(24), Article 16192. | Case report/series |
| 1. Biological and neuroimaging investigations | Genetics | 2022 | South Korea | Yang et al. | PSEN2 Thr421Met mutation in a patient with early onset Alzheimer’s disease | International Journal of Molecular Sciences, 23(21), Article 13331. | Case report/series |
| 1. Biological and neuroimaging investigations | Genetics | 2022 | Vietnam | Tong et al. | Genetic analysis of Vietnamese patients with early-onset Alzheimer's disease | International Journal of Neuroscience, 132(12), 1190–1197. | Cross-sectional/Survey |
| 1. Biological and neuroimaging investigations | Genetics | 2023 | China | Cai et al. | A pedigree study on early ⁃ onset Alzheimer's disease associated with PSEN2 V214L mutation, [伴 PSEN2基因 V214L 突变的早发型阿尔茨海默病一家系临床研究] | Zhongguo xian dai shen jing ji bing za zhi, 23(4), 335–340. | Case report/series |
| 1. Biological and neuroimaging investigations | Genetics | 2023 | China | Cheng et al. | Identification and functional characterization of novel variants of MAPT and GRN in Chinese patients with frontotemporal dementia | Neurobiology of Aging, 123, 233–243. | Cross-sectional/Survey |
| 1. Biological and neuroimaging investigations | Genetics | 2023 | China | Li et al. | A pedigree of early-onset familial Alzheimer's disease type 3 with spastic paraplegia as the primary manifestation, [以痉挛性截瘫为首发症状的早发型家族性阿尔茨海默病 3 型一家系] | Zhongguo xian dai shen jing ji bing za zhi, 23(9), 853–858. | Case report/series |
| 1. Biological and neuroimaging investigations | Genetics | 2023 | China | You et al. | Behavioural variant frontotemporal dementia due to CCNF gene mutation: A case report | Current Alzheimer Research, 20(5), 371–378. | Case report/series |
| 1. Biological and neuroimaging investigations | Genetics | 2023 | India | Sadhukhan et al. | An Indian young-onset dementia with parkinsonism with double heterozygous mutations in ABCA7 and PRKN identified through whole-exome sequencing | Alzheimer Disease & Associated Disorders,37(2),164-167. | Case report/series |
| 1. Biological and neuroimaging investigations | Genetics | 2023 | Japan | Mori et al. | A heterozygous splicing variant IVS9-7A > T in intron 9 of the MAPT gene in a patient with right-temporal variant frontotemporal dementia with atypical 4 repeat tauopathy | Acta Neuropathologica Communications, 11(1), Article 130. | Case report/series |
| 1. Biological and neuroimaging investigations | Genetics | 2023 | New Zealand | Ryan et al. | The New Zealand Genetic Frontotemporal Dementia Study (FTDGeNZ): a longitudinal study of pre-symptomatic biomarkers | Journal of the Royal Society of New Zealand, 53(4), 511–531. | Cohort |
| 1. Biological and neuroimaging investigations | Genetics | 2023 | Singapore | Tan et al. | C9orf72 expansions are the most common cause of genetic frontotemporal dementia in a Southeast Asian cohort | Annals of Clinical and Translational Neurology, 10(4), 568–578. | Cohort |
| 1. Biological and neuroimaging investigations | Genetics | 2023 | South Korea | Bae et al. | Double mutations in a patient with early-onset Alzheimer’s disease in Korea: An APP Val551Met and a PSEN2 His169Asn | International Journal of Molecular Sciences, 24(8), 7446. | Case report/series |
| 1. Biological and neuroimaging investigations | Genetics | 2023 | South Korea | Yang et al. | Patient with PSEN1 Glu318Gly and other possible disease risk mutations, diagnosed with early onset Alzheimer’s disease | International Journal of Molecular Sciences, 24(20), 15461. | Case report/series |
| 1. Biological and neuroimaging investigations | Genetics | 2023 | South Korea | Kang et al. | Effects of the APOE ϵ4 allele on the relationship between tau and amyloid-β in early- and late-onset Alzheimer's disease | Journal of Alzheimer's disease : JAD, 94(3), 1233–1246. | Case control |
| 1. Biological and neuroimaging investigations | Genetics | 2024 | China | Chang et al. | Case report: Double mutations in a patient with early-onset Alzheimer’s disease in China, PSEN2 and IDE variants | Frontiers in Neuroscience, 18, 1423892. | Case report/series |
| 1. Biological and neuroimaging investigations | multimodal investigations | 2010 | South Korea | Kim et al. | Comparison of neuropsychological and FDG-PET findings between early- versus late-onset mild cognitive impairment: A five-year longitudinal study | Dementia and Geriatric Cognitive Disorders, 29(3), 213–223. | Case control |
| 1. Biological and neuroimaging investigations | Multimodal investigations | 2011 | South Korea | Seo et al. | Effects of demographic factors on cortical thickness in Alzheimer's disease | Neurobiology of Aging, 32(2), 200–209. | Case control |
| 1. Biological and neuroimaging investigations | Multimodal investigations | 2017 | Japan | Kawakatsu et al. | Typical and atypical appearance of early onset Alzheimer’s disease: A clinical, neuroimaging and neuropathological study | Neuropathology, 37(2), 150–173. | Cohort |
| 1. Biological and neuroimaging investigations | Multimodal investigations | 2021 | China | Mao et al. | White matter hyperintensities and patterns of atrophy in early onset Alzheimer's disease with causative gene mutations | Clinical Neurology and Neurosurgery, 203, Article 106552. | Case report/series |
| 1. Biological and neuroimaging investigations | Multimodal investigations | 2022 | Singapore | Vipin et al. | Amyloid-tau-neurodegeneration profiles and longitudinal cognition in sporadic young-onset dementia | Journal of Alzheimer’s Disease, 90(2), 543–551. | Cohort |
| 1. Biological and neuroimaging investigations | Multimodal investigations | 2023 | China | Liang et al. | Clinical and genetic characteristics in a central-southern Chinese cohort of early-onset Alzheimer's disease | Frontiers in Neurology, 14, 1119326. | Cohort |
| 1. Biological and neuroimaging investigations | Multimodal investigations | 2024 | India | Gupta et al. | Utility of Tau PET in the diagnostic work up of neurodegenerative dementia among Indian patients | Journal of the Neurological Sciences, 467, Article 123292. | Cross-sectional/Survey |
| 1. Biological and neuroimaging investigations | Neuroimaging | 1983 | Japan | Arai et al. | A computed tomography study of Alzheimer's disease | Psychogeriatrics, 15(4), 255–271. | Cohort |
| 1. Biological and neuroimaging investigations | Neuroimaging | 1992 | Taiwan | Liu et al. | Single photon emission computed tomography using99Tcm-HMPAO in Alzheimer's disease | Nuclear Medicine Communications, 13(7), 535–541. | Case control |
| 1. Biological and neuroimaging investigations | Neuroimaging | 1994 | Japan | Kono et al. | Comparative study of cerebral ventricular dilation and cognitive function in patients with Alzheimer's disease of early versus late onset | Journal of Geriatric Psychiatry and Neurology, 7(1), 39–45. | Case control |
| 1. Biological and neuroimaging investigations | Neuroimaging | 1994 | Japan | Ichimiya et al. | Difference of regional cerebral metabolic pattern between presenile and senile dementia of the Alzheimer type: A factor analytic study | Journal of the Neurological Sciences, 123(1), 11–17 | Cross-sectional/Survey |
| 1. Biological and neuroimaging investigations | Neuroimaging | 2000 | Japan | Yamauchi et al. | Comparison of the pattern of atrophy of the corpus callosum in frontotemporal dementia, progressive supranuclear palsy, and Alzheimer's disease | Journal of Neurology, Neurosurgery & Psychiatry, 69(5), 623–629. | Case control |
| 1. Biological and neuroimaging investigations | Neuroimaging | 2000 | Japan | Shinotoh et al. | Progressive loss of cortical acetylcholinesterase activity in association with cognitive decline in Alzheimer's disease: A positron emission tomography study | Annals of Neurology, 48(2), 194–200. | Case control |
| 1. Biological and neuroimaging investigations | Neuroimaging | 2002 | Japan | Sakamoto et al. | Differences in cerebral metabolic impairment between early and late onset types of Alzheimer's disease | Journal of the Neurological Sciences, 200(1), 27–32. | Case control |
| 1. Biological and neuroimaging investigations | Neuroimaging | 2005 | South Korea | Kim et al. | Glucose metabolism in early onset versus late onset Alzheimer's disease: An SPM analysis of 120 patients | Brain (London, England : 1878), 128(8), 1790–1801. | Case control |
| 1. Biological and neuroimaging investigations | Neuroimaging | 2006 | Japan | Shiino et al. | Four subgroups of Alzheimer's disease based on patterns of atrophy using VBM and a unique pattern for early onset disease | NeuroImage (Orlando, Fla.), 33(1), 17–26. | Case control |
| 1. Biological and neuroimaging investigations | Neuroimaging | 2008 | Japan | Shiino et al. | Different atrophic patterns in early- and late-onset Alzheimer's disease and evaluation of clinical utility of a method of regional z-score analysis using voxel-based morphometry | Dementia and Geriatric Cognitive Disorders, 26(2), 175–186. | Case control |
| 1. Biological and neuroimaging investigations | Neuroimaging | 2009 | Australia | Panegyres et al. | Fluorodeoxyglucose-positron emission tomography in the differential diagnosis of early-onset dementia: A prospective, community-based study | BMC Neurology, 9(1), Article 41. | Cross-sectional/Survey |
| 1. Biological and neuroimaging investigations | Neuroimaging | 2009 | Japan | Kiuchi et al. | Abnormalities of the uncinate fasciculus and posterior cingulate fasciculus in mild cognitive impairment and early Alzheimer's disease: A diffusion tensor tractography study | Brain Research, 1287, 184–191. | Case control |
| 1. Biological and neuroimaging investigations | Neuroimaging | 2009 | Japan | Mitsumoto et al. | Diagnostic performance of Tc-99m HMPAO SPECT for early and late onset Alzheimer's disease: A clinical evaluation of linearization correction | Annals of Nuclear Medicine, 23(5), 487–495. | Case control |
| 1. Biological and neuroimaging investigations | Neuroimaging | 2011 | South Korea | Choo et al. | Relationship of amyloid-beta burden with age-at-onset in alzheimer disease | The American Journal of Geriatric Psychiatry, 19(7), 627–634. | Cross-sectional/Survey |
| 1. Biological and neuroimaging investigations | Neuroimaging | 2012 | India | Kumari et al. | Segmentation of MR brain images using FCM technique in Frontotemporal Dementia | IET Chennai 3rd International Conference on Sustainable Energy and Intelligent Systems (SEISCON 2012), 62–65. | Case control |
| 1. Biological and neuroimaging investigations | Neuroimaging | 2013 | Japan | Ito et al. | Massive accumulation of 11C-Pittsburg compound B in the occipital lobes of a patient with early-onset dementia accompanied by muscle weakness and hypertonicity | Annals of Nuclear Medicine, 27(10), 935–941. | Case report/series |
| 1. Biological and neuroimaging investigations | Neuroimaging | 2013 | Japan | Terasawa et al. | Increasing and persistent DWI changes in a patient with hereditary diffuse leukoencephalopathy with spheroids | ournal of the Neurological Sciences, 335(1–2), 213–215. | Case report/series |
| 1. Biological and neuroimaging investigations | Neuroimaging | 2013 | South Korea | Cho et al. | Changes in subcortical structures in early- versus late-onset Alzheimer's disease | Neurobiology of Aging, 34(7), 1740–1747. | Case control |
| 1. Biological and neuroimaging investigations | Neuroimaging | 2013 | South Korea | Cho et al. | Longitudinal changes of cortical thickness in early- versus late-onset Alzheimer's disease | Neurobiology of Aging, 34(7), 1921.e9-1921.e15. | Case control |
| 1. Biological and neuroimaging investigations | Neuroimaging | 2015 | Japan | Fujisawa et al. | Alzheimer's disease or Alzheimer's syndrome?: A longitudinal computed tomography neuroradiological follow-up study of 56 cases diagnosed clinically as Alzheimer's disease | Psychogeriatrics, 15(4), 255–271. | Cohort |
| 1. Biological and neuroimaging investigations | Neuroimaging | 2016 | South Korea | Chung et al. | Glucose metabolic brain networks in early-onset vs. late-onset Alzheimer's disease | Frontiers in Aging Neuroscience, 8, 159. | Case control |
| 1. Biological and neuroimaging investigations | Neuroimaging | 2017 | South Korea | Cho et al. | Excessive tau accumulation in the parieto-occipital cortex characterizes early-onset Alzheimer's disease | Neurobiology of Aging, 53, 103–111. | Case control |
| 1. Biological and neuroimaging investigations | Neuroimaging | 2017 | South Korea | Park et al. | Functional connectivity of the hippocampus in early- and vs. late-onset Alzheimer's disease | Journal of Clinical Neurology (Seoul, Korea), 13(4), 387–393. | Case control |
| 1. Biological and neuroimaging investigations | Neuroimaging | 2018 | China | Li et al. | Distinct patterns of interhemispheric connectivity in patients with early- and late-onset Alzheimer’s disease | Frontiers in Aging Neuroscience, 10, 261. | Case control |
| 1. Biological and neuroimaging investigations | Neuroimaging | 2018 | China | Moon et al. | Changes in the hippocampal volume and shape in early-onset mild cognitive impairment | Psychiatry Investigation, 15(5), 531–537. | Cohort |
| 1. Biological and neuroimaging investigations | Neuroimaging | 2019 | Australia | Cherbuin et al. | Validated Alzheimer’s Disease Risk Index (ANU-ADRI) is associated with smaller volumes in the default mode network in the early 60s | Brain Imaging and Behavior, 13(1), 65–74. | Cross-sectional/Survey |
| 1. Biological and neuroimaging investigations | Neuroimaging | 2019 | India | Mukku et al. | Clinical utility of 18F-FDG-PET/MRI brain in dementia: Preliminary experience from a geriatric clinic in South India | Asian Journal of Psychiatry, 44, 99–105. | Cross-sectional/Survey |
| 1. Biological and neuroimaging investigations | Neuroimaging | 2019 | Taiwan | Huang et al. | Amyloid PET pattern with dementia and amyloid angiopathy in Taiwan familial AD with D678H APP mutation | Journal of the Neurological Sciences, 398, 107–116. | Cross-sectional/Survey |
| 1. Biological and neuroimaging investigations | Neuroimaging | 2020 | Japan | Chishiki et al. | Different patterns of gray matter volume reduction in early-onset and late-onset Alzheimer disease | Cognitive and behavioral neurology : official journal of the Society for Behavioral and Cognitive Neurology, 33(4), 253–258. | Case control |
| 1. Biological and neuroimaging investigations | Neuroimaging | 2020 | South Korea | San et al. | Differences in neuroimaging features of early- versus late-onset nonfluent/agrammatic primary progressive aphasia | Neurobiology of Aging, 86, 92–101. | Case control |
| 1. Biological and neuroimaging investigations | Neuroimaging | 2021 | China | Zhang et al. | Choriocapillaris changes are correlated with disease duration and MoCA score in early-onset dementia | Frontiers in Aging Neuroscience, 13, 656750. | Case control |
| 1. Biological and neuroimaging investigations | Neuroimaging | 2021 | China | Qin et al. | Prominent striatum amyloid retention in early-onset familial Alzheimer's disease with PSEN1 mutations: A pilot PET/MR study | Frontiers in Aging Neuroscience, 13, 732159. | Case control |
| 1. Biological and neuroimaging investigations | Neuroimaging | 2021 | Taiwan | Li et al. | Language impairment as diagnostic clue to lvPPA in a young-onset dementia patient mimicking bvFTD. | Australian & New Zealand Journal of Psychiatry, 55(2), 225–226. | Case report/series |
| 1. Biological and neuroimaging investigations | Neuroimaging | 2022 | China | Du et al. | Radiomic features of the hippocampus for diagnosing early-onset and late-onset Alzheimer's disease | Frontiers in Aging Neuroscience, 13, 789099. | Case control |
| 1. Biological and neuroimaging investigations | Neuroimaging | 2022 | Japan | Kobayashi et al. | Comparing medial temporal atrophy between early-onset semantic dementia and early-onset Alzheimer's disease using voxel-based morphometry: A multicenter MRI study | Current Alzheimer Research, 19(7), 503–510. | Cross-sectional/Survey |
| 1. Biological and neuroimaging investigations | Neuroimaging | 2023 | China | Luo et al. | Distinct cerebral small vessel disease impairment in early- and late-onset Alzheimer's disease | Annals of Clinical and Translational Neurology, 10(8), 1326–1337. | Case control |
| 1. Biological and neuroimaging investigations | Neuroimaging | 2024 | China | Kwapong et al. | Choriocapillaris reduction accurately discriminates against early-onset alzheimer's disease | Alzheimer’s & Dementia, 20(6), 4185–4198. | Case control |
| 1. Biological and neuroimaging investigations | Neuroimaging | 2024 | China | Chen et al. | Incremental value of amyloid PET in a tertiary memory clinic setting in China | Alzheimer’s & Dementia, 20(4), 2516–2525. | Cohort |
| 1. Biological and neuroimaging investigations | Neuroimaging | 2024 | Japan | Sato et al. | Characteristics of Alzheimer’s disease and mild cognitive impairment influenced by the time of onset | Dementia and Geriatric Cognitive Disorders Extra, 14(1), 81–88. | Case control |
| 1. Biological and neuroimaging investigations | Neuroimaging | 2024 | South Korea | Heo et al. | Alterations of structural network efficiency in early-onset and late-onset Alzheimer’s disease | Journal of Clinical Neurology (Seoul, Korea), 20(3), 265–275. | Case control |
| 1. Biological and neuroimaging investigations | Neuroimaging | 2024 | South Korea | Na et al. | Diverging relationships among amyloid, tau, and brain atrophy in early-onset and late-onset Alzheimer’s disease | Yonsei Medical Journal, 65(8), 434–447. | Case control |
| 1. Biological and neuroimaging investigations | Neuroimaging | 2024 | South Korea | Jung et al. | Comparison of enlarged perivascular spaces in early-onset and late-onset Alzheimer disease-related cognitive impairment: A single clinic-based study in South Korea | Alzheimer disease and associated disorders, 38(2), 201–204. | Cross-sectional/Survey |
| 1. Biological and neuroimaging investigations | Neuroimaging | 2024 | Vietnam | Nguyen et al. | Lateral temporal atrophy is a better predictor of baseline MMSE scores than hippocampal atrophy in Alzheimer’s disease: A retrospective cross-sectional study | Neurology Asia, 29(2), 421–429. | Cross-sectional/Survey |
| 2. Clinical features and course | Clinical features and course | 1984 | Japan | Mitsuyama et al. | Presenile dementia with motor neuron disease in Japan: Clinico-pathological review of 26 cases | Journal of Neurology, Neurosurgery and Psychiatry, 47(9), 953–959. | Case report/series |
| 2. Clinical features and course | Clinical features and course | 1987 | Japan | Morita et al. | Presenile dementia combined with amyotrophy: A review of 34 Japanese case | Arch Gerontol Geriatr, 6(3), 263–277. | Case report/series |
| 2. Clinical features and course | Clinical features and course | 1989 | Taiwan | Fink et al. | Clinical spectrum of niemann-pick disease type C | Neurology, 39(8), 1040–1040. | Cohort |
| 2. Clinical features and course | Clinical features and course | 1997 | Japan | Ishikawa et al. | Clinical features of familial diffuse lewy body disease | European Neurology, 38(Suppl 1), 34–38. | Case report/series |
| 2. Clinical features and course | Clinical features and course | 1998 | China | Wei et al. | Results of Wechsler Memory Scale in pre-senile and senile subjects | Chinese Mental Health Journal, 12(6), 340-341. | Cross-sectional/Survey |
| 2. Clinical features and course | Clinical features and course | 1999 | Japan | Takagi et al. | Sensitive pupil response of early-onset alzheimer's patients to a dilute mixture of cholinergic antagonist and α-adrenergic stimulant | Environmental Health and Preventive Medicine, 4(1), 49–53. | Case control |
| 2. Clinical features and course | Clinical features and course | 2004 | Australia | Piguet et al. | Similar early clinical presentations in familial and non-familial frontotemporal dementia | Journal of Neurology, Neurosurgery and Psychiatry, 75(12), 1743–1745. | Cross-sectional/Survey |
| 2. Clinical features and course | Clinical features and course | 2004 | India | Sundar et al. | Presenile dementia - Etiology, clinical profile and treatment response at four month follow up | The Journal of the Association of Physicians of India, 52, 953–958. | Cohort |
| 2. Clinical features and course | Clinical features and course | 2005 | Japan | Hori et al. | First episodes of behavioral symptoms in Alzheimer's disease patients at age 90 and over, and early-onset Alzheimer's disease: Comparison with senile dementia of Alzheimer's type | Psychiatry and Clinical Neurosciences, 59(6), 730–735. | Cross-sectional/Survey |
| 2. Clinical features and course | Clinical features and course | 2005 | Japan | Yokota et al. | Frequency of early and late-onset dementias in a Japanese memory disorders clinic | European Journal of Neurology, 12(10), 782–790. | Cross-sectional/Survey |
| 2. Clinical features and course | Clinical features and course | 2005 | Japan | Shinagawa et al. | Initial symptoms in frontotemporal dementia and semantic dementia compared with Alzheimer’s disease | Dementia and Geriatric Cognitive Disorders, 21(2), 74–80. | Cross-sectional/Survey |
| 2. Clinical features and course | Clinical features and course | 2005 | Japan | Maeda et al. | Case of pseudo-pseudohypoparathyroidism associated with juvenile dementia | Psychiatry and Clinical Neurosciences, 59(1), 111–111. | Case report/series |
| 2. Clinical features and course | Clinical features and course | 2006 | India | Mathuranath et al. | Profiles of language impairment in progressive nonfluent aphasia | Annals of the Indian Academy of Neurology, 9(1), 25. | Case report/series |
| 2. Clinical features and course | Clinical features and course | 2007 | Australia | Panegyres et al. | Course and causes of suspected dementia in young adults: A longitudinal study | American Journal of Alzheimer’s Disease and Other Dementias, 22(1), 48–56. | Cohort |
| 2. Clinical features and course | Clinical features and course | 2007 | Australia | Panegyres et al. | The clinical differentiation of fronto-temporal dementia from psychiatric disease | Neuropsychiatric Disease and Treatment, 3(5), 637–645. | Case control |
| 2. Clinical features and course | Clinical features and course | 2007 | Japan | Toyota et al. | Comparison of behavioral and psychological symptoms in early-onset and late-onset Alzheimer's disease | International Journal of Geriatric Psychiatry, 22(9), 896–901. | Cross-sectional/Survey |
| 2. Clinical features and course | Clinical features and course | 2007 | Japan | Shinagawa et al. | Frequency and clinical characteristics of early-onset dementia in consecutive patients in a memory clinic | Dementia and Geriatric Cognitive Disorders, 24(1), 42–47. | Cross-sectional/Survey |
| 2. Clinical features and course | Clinical features and course | 2008 | India | Nandi et al. | Clinical profile of young-onset dementia: A study from Eastern India | Neurology Asia, 13, 103-108. | Cohort |
| 2. Clinical features and course | Clinical features and course | 2008 | Japan | Shinagawa et al. | Cognitive function and psychiatric symptoms in early- and late-onset frontotemporal dementia | Dementia and Geriatric Cognitive Disorders, 25(5), 439–444. | Cross-sectional/Survey |
| 2. Clinical features and course | Clinical features and course | 2009 | Australia | Omar et al. | Delusions in frontotemporal lobar degeneration | Journal of Neurology, 256(4), Article 600. | Case report/series |
| 2. Clinical features and course | Clinical features and course | 2011 | Australia | Fairjones et al. | Exploring the role of cognitive reserve in early-onset dementia | American Journal of Alzheimer’s Disease and Other Dementias, 26(2), 139–144. | Case control |
| 2. Clinical features and course | Clinical features and course | 2011 | Australia | Brodaty et al. | Patients in Australian memory clinics: Baseline characteristics and predictors of decline at six months | International Psychogeriatrics, 23(7), 1086–1096. | Cohort |
| 2. Clinical features and course | Clinical features and course | 2011 | China | Chong et al. | Presenile dementia: A case of Hashimoto's encephalopathy | East Asian Archives of Psychiatry, 21(1), 32–36. | Case report/series |
| 2. Clinical features and course | Clinical features and course | 2011 | Hong Kong | Liu et al. | A case of young-onset dementia | Hong Kong Medical Journal = Xianggang Yi Xue Za Zhi, 17(3), 248. | Case report/series |
| 2. Clinical features and course | Clinical features and course | 2011 | India | Alladi et al. | Subtypes of dementia: A study from a memory clinic in India | Dementia and Geriatric Cognitive Disorders, 32(1), 32–38. | Cross-sectional/Survey |
| 2. Clinical features and course | Clinical features and course | 2011 | Japan | Nagata et al. | Association between executive dysfunction and hippocampal volume in Alzheimer's disease | International Psychogeriatrics, 23(5), 764–771. | Cross-sectional/Survey |
| 2. Clinical features and course | Clinical features and course | 2011 | Japan | Shimizu et al. | Clinical profiles of late-onset semantic dementia, compared with early-onset semantic dementia and late-onset Alzheimer's disease | Psychogeriatrics, 11(1), 46–53. | Cross-sectional/Survey |
| 2. Clinical features and course | Clinical features and course | 2011 | Japan | Tsunoda et al. | Presenile dementia diagnosed as posterior cortical atrophy | Psychogeriatrics, 11(3), 171–176. | Case report/series |
| 2. Clinical features and course | Clinical features and course | 2011 | South Korea | Yoon et al. | Agraphia in Korean patients with early onset Alzheimer's disease. | International Psychogeriatrics, 23(8), 1317–1326. | Case control |
| 2. Clinical features and course | Clinical features and course | 2012 | Australia | Atkins et al. | The natural history of early-onset dementia: The Artemis Project | BMJ Open, 2(5), e001764. | Cohort |
| 2. Clinical features and course | Clinical features and course | 2013 | China | Mu et al. | Study of lipids, insulin metabolism, and paraoxonase-2-311 polymorphism in patients with different subtypes of Alzheimer's disease (translated version) | East Asian Archives of Psychiatry, 23(3), 114–119. | Cross-sectional/Survey |
| 2. Clinical features and course | Clinical features and course | 2013 | Japan | Funayama et al. | Progression of logopenic variant primary progressive aphasia to apraxia and semantic memory deficits | BMC Neurology, 13(1), Article 158. | Case report/series |
| 2. Clinical features and course | Clinical features and course | 2013 | Japan | Hamuro | Combination therapy with galantamine and memantine improves behavioral and psychological symptoms of dementia (BPSD) in patients with early-onset Alzheimer’s disease | Australian and New Zealand Journal of Psychiatry, 47(6), 583–583. | Case report/series |
| 2. Clinical features and course | Clinical features and course | 2013 | Taiwan | Pai et al. | Impaired translation of spatial representation in young onset Alzheimer's disease patients | Current Alzheimer Research, 10(1), 95–103. | Case control |
| 2. Clinical features and course | Clinical features and course | 2014 | Australia | Panegyres et al. | Early-onset Alzheimer's disease: A global cross-sectional analysis | European Journal of Neurology, 21(9), 1149-e65. | Cross-sectional/Survey |
| 2. Clinical features and course | Clinical features and course | 2014 | China | Zhao et al. | Cognitive decline in patients with Alzheimer's disease and its related factors in a memory clinic setting, Shanghai, China | PloS One, 9(4), e95755. | Cohort |
| 2. Clinical features and course | Clinical features and course | 2014 | Japan | Ota et al. | Three presenile patients in which neuropsychological and neuroimaging examinations suggest possible progression to dementia with Lewy bodies | Psychogeriatrics, 14(1), 72–80. | Case report/series |
| 2. Clinical features and course | Clinical features and course | 2014 | Japan | Yamakawa et al. | Caring for early-onset dementia with excessive wandering of over 30 kilometres per day: A case report | Psychogeriatrics, 14(4), 255–260. | Case report/series |
| 2. Clinical features and course | Clinical features and course | 2014 | Taiwan | Lin et al. | Association of early-onset dementia with activities of daily living (ADL) in middle-aged adults with intellectual disabilities: The caregiver's perspective | Research in Developmental Disabilities, 35(3), 626–631. | Cross-sectional/Survey |
| 2. Clinical features and course | Clinical features and course | 2014 | Taiwan | Goh et al. | Hashimoto's encephalopathy mimicking presenile dementia | General Hospital Psychiatry, 36(3), 360.e9-360.e11. | Case report/series |
| 2. Clinical features and course | Clinical features and course | 2015 | Japan | Tanaka et al. | Relationship between dementia severity and behavioural and psychological symptoms in early-onset Alzheimer's disease | Psychogeriatrics, 15(4), 242–247. | Cross-sectional/Survey |
| 2. Clinical features and course | Clinical features and course | 2015 | South Korea | Park et al. | Cognitive profiles and neuropsychiatric symptoms in Korean early-onset Alzheimer's disease patients: A CREDOS study | Journal of Alzheimer’s Disease, 44(2), 661–673. | Cross-sectional/Survey |
| 2. Clinical features and course | Clinical features and course | 2015 | South Korea | Ye et al. | Clinical and neuropsychological comparisons of early-onset versus late-onset frontotemporal dementia: a CREDOS-FTD study | Journal of Alzheimer’s Disease, 45(2), 599–608. | Case control |
| 2. Clinical features and course | Clinical features and course | 2015 | South Korea | Yoon et al. | Predictive factors for disease progression in patients with early-onset Alzheimer's disease | Journal of Alzheimer’s Disease, 49(1), 85–91. | Cohort |
| 2. Clinical features and course | Clinical features and course | 2015 | Thailand | Carr et al. | An investigation of care-based vs. rule-based morality in frontotemporal dementia, Alzheimer's disease, and healthy controls | Neuropsychologia, 78, 73–79. | Case control |
| 2. Clinical features and course | Clinical features and course | 2016 | India | Chandra et al. | Neuropsychiatric symptoms in a cohort of patients with frontotemporal dementia: Our experience | Indian Journal of Psychological Medicine, 38(4), 326–330. | Cohort |
| 2. Clinical features and course | Clinical features and course | 2016 | Japan | Yoshimura et al. | A comparative analysis of dementia inpatient characteristics: Results from a nationwide survey of different care facilities in Japan | Psychogeriatrics, 16(1), 34–45. | Cross-sectional/Survey |
| 2. Clinical features and course | Clinical features and course | 2016 | Japan | Midorikawa et al. | All is Not Lost: Positive Behaviors in Alzheimer's Disease and Behavioral-Variant Frontotemporal Dementia with Disease Severity | Journal of Alzheimer’s Disease, 54(2), 549–558. | Cross-sectional/Survey |
| 2. Clinical features and course | Clinical features and course | 2016 | South Korea | Yoon et al. | Differences in depressive patterns according to disease severity in early-onset Alzheimer's disease | Journal of Alzheimer’s Disease, 52(1), 91–99. | Cross-sectional/Survey |
| 2. Clinical features and course | Clinical features and course | 2016 | South Korea | Jang et al. | Early- vs late-onset subcortical vascular cognitive impairment | Neurology, 86(6), 527–534. | Cross-sectional/Survey |
| 2. Clinical features and course | Clinical features and course | 2017 | Hong Kong | Shea et al. | The first case series of chinese patients in Hong Kong with familial Alzheimer’s disease compared with those with biomarker-confirmed sporadic late-onset Alzheimer’s disease | Hong Kong Medical Journal = Xianggang Yi Xue Za Zhi, 23(6), 579. | Case report/series |
| 2. Clinical features and course | Clinical features and course | 2017 | India | Chandra et al. | The tale of the storyteller and the painter: The paradoxes in nature | Indian Journal of Psychological Medicine, 39(6), 817–820. | Case report/series |
| 2. Clinical features and course | Clinical features and course | 2017 | India | Singh et al. | Neurosyphilis: An underdiagnosed entity. | Asian Journal of Psychiatry, 30, 47–48. | Case report/series |
| 2. Clinical features and course | Clinical features and course | 2017 | South Korea | Yoon et al. | Anosognosia and its relation to psychiatric symptoms in early-onset Alzheimer disease | Journal of Geriatric Psychiatry and Neurology, 30(3), 170–177. | Cross-sectional/Survey |
| 2. Clinical features and course | Clinical features and course | 2018 | Australia | Hutchings et al. | Looking but not seeing: Increased eye fixations in behavioural-variant frontotemporal dementia | Cortex, 103, 71–81. | Case control |
| 2. Clinical features and course | Clinical features and course | 2018 | India | Mukku et al. | Posterior cortical atrophy variant of Alzheimer's dementia-A case report | Asian Journal of Psychiatry, 35, 109–112. | Case report/series |
| 2. Clinical features and course | Clinical features and course | 2018 | Japan | Kobayashi et al. | Clinical features of the behavioural variant of frontotemporal dementia that are useful for predicting underlying pathological subtypes of frontotemporal lobar degeneration | Psychogeriatrics, 18(4), 307–312. | Cross-sectional/Survey |
| 2. Clinical features and course | Clinical features and course | 2018 | Japan | Aiba et al. | Young-onset dementia with lewy bodies | Case Reports in Neurology, 10(3), 363–368. | Case report/series |
| 2. Clinical features and course | Clinical features and course | 2018 | New Zealand | Payman et al. | Early onset dementia in New Zealand Pacific boxers: A case series | New Zealand Medical Journal, 131(1474), 20–26. | Case report/series |
| 2. Clinical features and course | Clinical features and course | 2019 | Australia | Wong et al. | Frontal variant of Alzheimer's disease masquerading as behavioural-variant frontotemporal dementia: A case study comparison | Neurocase, 25(1–2), 48–58. | Case report/series |
| 2. Clinical features and course | Clinical features and course | 2019 | China | Wang et al. | Treatable causes of adult-onset rapid cognitive impairment | Clinical Neurology and Neurosurgery, 187, Article 105575. | Cohort |
| 2. Clinical features and course | Clinical features and course | 2019 | Hong Kong | Au et al. | Early-onset dementia in chinese: Demographic and etiologic characteristics | Neurology Asia, 24(2), 139–146. | Cross-sectional/Survey |
| 2. Clinical features and course | Clinical features and course | 2020 | China | Cai et al. | Patient with frontal-variant syndrome in early-onset Alzheimer's disease | General Psychiatry, 33(2), e100173. | Case report/series |
| 2. Clinical features and course | Clinical features and course | 2020 | China | Li et al. | Case report of first-episode psychotic symptoms in a patient with early-onset Alzheimer's disease | BMC Psychiatry, 20(1), Article 128. | Case report/series |
| 2. Clinical features and course | Clinical features and course | 2020 | Japan | Sasaki et al. | Late-onset attention-deficit/hyperactivity disorder as a differential diagnosis of dementia: A case report | BMC Psychiatry, 20(1), Article 550. | Case report/series |
| 2. Clinical features and course | Clinical features and course | 2021 | Australia | Loi et al. | A 10 year retrospective cohort study of inpatients with younger-onset dementia | International Journal of Geriatric Psychiatry, 36(2), 294–301. | Cohort |
| 2. Clinical features and course | Clinical features and course | 2021 | Australia | Panegyres | The clinical spectrum of young onset dementia points to its stochastic origins | JAD Reports, 5(1), 663–679. | Cohort |
| 2. Clinical features and course | Clinical features and course | 2021 | Australia | Wang et al. | Predictors of new-onset epilepsy in people with younger-onset neurocognitive disorders | Frontiers in Aging Neuroscience, 13, 637260. | Case control |
| 2. Clinical features and course | Clinical features and course | 2021 | Japan | Hirano et al. | Characteristics of early-onset dementia in Chiba Prefecture, Japan: A multicenter survey | Dementia and Geriatric Cognitive Disorders, 50(3), 283–288. | Cross-sectional/Survey |
| 2. Clinical features and course | Clinical features and course | 2021 | New Zealand | Ryan et al. | Sociodemographic and clinical characteristics of 1350 patients with young onset dementia: A comparison with older patients | Alzheimer disease and associated disorders, 35(3), 200–207. | Cross-sectional/Survey |
| 2. Clinical features and course | Clinical features and course | 2021 | Singapore | Vipin et al. | Dementia in Southeast Asia: influence of onset-type, education, and cerebrovascular disease | Alzheimer’s Research & Therapy, 13(1), Article 195. | Cohort |
| 2. Clinical features and course | Clinical features and course | 2021 | South Korea | Sohn et al. | A case of CSF1R-related leukoencephalopathy: serial neuroimaging and neuropsychological tests | Neurocase, 27(5), 415–418. | Case report/series |
| 2. Clinical features and course | Clinical features and course | 2021 | Thailand | Dharmasaroja et al. | Etiology of dementia in Thai patients | Dementia and Geriatric Cognitive Disorders Extra, 11(1), 64–70. | Cohort |
| 2. Clinical features and course | Clinical features and course | 2022 | Australia | Loi et al. | Clinico-demographics of people with younger-onset dementia and neuropsychiatric symptoms referred to an Australian dementia support service: A comparison study with older-onset dementia | Australian and New Zealand Journal of Psychiatry, 56(12), 1653–1663. | Cross-sectional/Survey |
| 2. Clinical features and course | Clinical features and course | 2022 | China | Fang et al. | Behavioural and psychological symptoms of early-onset and late-onset alzheimer's disease among chinese adults: Analysis of modifiable factors | Psychogeriatrics, 22(3), 391–401. | Cross-sectional/Survey |
| 2. Clinical features and course | Clinical features and course | 2022 | China | Mi et al. | Capgras syndrome as the core manifestation of early-onset Alzheimer's disease | Journal of Alzheimer’s Disease, 87(1), 155–160. | Case report/series |
| 2. Clinical features and course | Clinical features and course | 2022 | China | Zhang et al. | Microvascular changes in the retina correlate with mri markers in patients with early-onset dementia | Brain Sciences, 12(10), 1391. | Cross-sectional/Survey |
| 2. Clinical features and course | Clinical features and course | 2022 | India | Parihar et al. | Vanishing white matter disease presenting as dementia and infertility: A case report | Neurology. Genetics, 8(3), e643. | Case report/series |
| 2. Clinical features and course | Clinical features and course | 2022 | India | Krishnadas et al. | Nasu-Hakola disease - A rare type of presenile dementia. | Annals of the Indian Academy of Neurology, 25(4), 771–772. | Case report/series |
| 2. Clinical features and course | Clinical features and course | 2022 | Singapore | Sim et al. | Clinical manifestations of early-onset dementia with Lewy bodies compared with late-onset dementia with Lewy bodies and early-onset Alzheimer disease | JAMA Neurology, 79(7), 702–709. | Case control |
| 2. Clinical features and course | Clinical features and course | 2022 | South Korea | Kim et al. | Eye-tracking metrics for figure-copying processes in early- vs. late-onset Alzheimer's disease | Frontiers in Neurology, 13, 844341. | Case control |
| 2. Clinical features and course | Clinical features and course | 2023 | Australia | Borelli-Millott et al. | A thematic analysis of psychotic symptoms in young-onset dementia | International Psychogeriatrics, 36(6), 465–473. | Qualitative |
| 2. Clinical features and course | Clinical features and course | 2023 | China | Jia et al. | A 19-year-old adolescent with probable Alzheimer's disease | Journal of Alzheimer’s Disease, 91(3), 915. | Case report/series |
| 2. Clinical features and course | Clinical features and course | 2023 | Hong Kong | Ip et al. | A curious case of early-onset dementia | Hong Kong medical journal = Xianggang yi xue za zhi, 29(4), 359.e1–359.e3. | Case report/series |
| 2. Clinical features and course | Clinical features and course | 2023 | India | Mahale et al. | Young-onset Alzheimer’s dementia mimicking progressive myoclonic epilepsy spectrum | The Egyptian Journal of Neurology, Psychiatry and Neurosurgery, 59(1), 140–143. | Case report/series |
| 2. Clinical features and course | Clinical features and course | 2023 | Japan | Edahiro et al. | Initial symptoms of early-onset dementia in Japan: Nationwide survey | Psychogeriatrics, 23(3), 422–433. | Cross-sectional/Survey |
| 2. Clinical features and course | Clinical features and course | 2023 | Malaysia | Abdullah et al. | It is not depression: A case report of a 37-year-old firefighter with frontotemporal dementia | Malaysian Family Physician, 18, 46–5. | Case report/series |
| 2. Clinical features and course | Clinical features and course | 2023 | South Korea | Kim et al. | Motor symptoms in early- versus late-onset Alzheimer's disease | Journal of Alzheimer’s Disease, 91(1), 345–354. | Cross-sectional/Survey |
| 2. Clinical features and course | Clinical features and course | 2023 | Sri Lanka | Hewawasam et al. | Subclinical memory impairment in unaffected siblings of patients with dementia | Clinical Neuropsychologist, 37(8), 1669–1685. | Case control |
| 2. Clinical features and course | Clinical features and course | 2024 | Australia | Reutens et al. | Characteristics of domestic violence perpetrators with dementia from police records using text mining | Frontiers in Psychiatry, 15, 1331915. | Cross-sectional/Survey |
| 2. Clinical features and course | Clinical features and course | 2024 | China | Shang et al. | Comorbidity of dementia: A cross-sectional study of PUMCH dementia cohort | Journal of Alzheimer’s Disease, 97(3), 1313–1322. | Cross-sectional/Survey |
| 2. Clinical features and course | Clinical features and course | 2024 | India | Vijay et al. | A Rare Case of Very Young-Onset Dementia Presenting as Conversion Disorder | Medical Journal of Dr. D Y Patil University, 17(3), 671–672. | Case report/series |
| 2. Clinical features and course | Clinical features and course | 2024 | Japan | Matsumoto et al. | Early-onset dementia and risk of hip fracture and major osteoporotic fractures | Alzheimer’s & Dementia, 20(5), 3388–3396. | Case control |
| 3. Epidemiology and outcomes | Combined epidemiological studies | 2017 | Australia | Davis et al. | Dementia onset, incidence and risk in type 2 diabetes: a matched cohort study with the Fremantle Diabetes Study Phase I | Diabetologia, 60(1), 89–97. | Cohort |
| 3. Epidemiology and outcomes | Combined epidemiological studies | 2020 | South Korea | Yu et al. | Incidence and risk factors for dementia in type 2 diabetes mellitus: A nationwide population-based study in Korea | Diabetes & Metabolism Journal, 44(1), 113–124. | Cohort |
| 3. Epidemiology and outcomes | Combined epidemiological studies | 2024 | South Korea | Chun et al. | Effects of risk factors on the development and mortality of early- and late-onset dementia: an 11-year longitudinal nationwide population-based cohort study in South Korea | Alzheimer’s Research & Therapy, 16(1), Article 92. | Cohort |
| 3. Epidemiology and outcomes | Etiology and risk factors | 1994 | Japan | Kurita et al. | A young case with multi-infarct dementia associated with lupus anticoagulant | Internal Medicine, 33(6), 373–375. | Case report/series |
| 3. Epidemiology and outcomes | Etiology and risk factors | 2012 | Australia | Atkins et al. | Cerebrovascular risk factors in early-onset dementia | Journal of Neurology, Neurosurgery and Psychiatry, 83(6), 666. | Cross-sectional/Survey |
| 3. Epidemiology and outcomes | Etiology and risk factors | 2013 | Japan | Ueda et al. | Neuronal ceroid lipofuscinosis with early-onset dementia and periventricular leukoencephalopathy in which a skin biopsy was diagnostically useful | Internal Medicine, 52(19), 2271–2274. | Case report/series |
| 3. Epidemiology and outcomes | Etiology and risk factors | 2014 | India | Verma et al. | HIV presenting as young-onset dementia | Journal of the International Association of Providers of AIDS Care, 13(2), 110–112. | Case report/series |
| 3. Epidemiology and outcomes | Etiology and risk factors | 2017 | India | Korada et al. | Personality traits in the siblings and children of patients with frontotemporal dementia: A questionnaire-based study | Indian Journal of Psychological Medicine, 39(1), 28–31. | Case control |
| 3. Epidemiology and outcomes | Etiology and risk factors | 2017 | Japan | Kadohara et al. | Diabetes mellitus and risk of early-onset Alzheimer's disease: a population-based case–control study | European Journal of Neurology, 24(7), 944–949. | Case control |
| 3. Epidemiology and outcomes | Etiology and risk factors | 2018 | Australia | Cations et al. | Non-genetic risk factors for degenerative and vascular young onset dementia: Results from the INSPIRED and KGOW studies | Journal of Alzheimer’s Disease, 62(4), 1747–1758. | Case control |
| 3. Epidemiology and outcomes | Etiology and risk factors | 2019 | India | Lahiri et al. | Young-onset sporadic Creutzfeldt-Jakob disease with atypical phenotypic features: A case report | Journal of Medical Case Reports, 13(1), Article 163. | Case report/series |
| 3. Epidemiology and outcomes | Etiology and risk factors | 2019 | Taiwan | Wu et al. | Memory complaint is a surrogate for memory decline in the middle-aged: A register-based study | Journal of Clinical Medicine, 8(11), 1900. | Cohort |
| 3. Epidemiology and outcomes | Etiology and risk factors | 2020 | Australia | Cations et al. | Clustering and additive effects of nongenetic risk factors in non-autosomal-dominant degenerative and vascular young onset dementia | Alzheimer disease and associated disorders, 34(2), 128–134. | Case control |
| 3. Epidemiology and outcomes | Etiology and risk factors | 2020 | South Korea | Han et al. | Gamma glutamyltransferase and risk of dementia in prediabetes and diabetes | Scientific Reports, 10(1), Article 6800. | Cohort |
| 3. Epidemiology and outcomes | Etiology and risk factors | 2020 | South Korea | Kim et al. | Atrial fibrillation increases the risk of early-onset dementia in the general population: Data from a population-based cohort | Journal of Clinical Medicine, 9(11), 3665. | Cohort |
| 3. Epidemiology and outcomes | Etiology and risk factors | 2021 | Australia | He et al. | Increased VLCFA-lipids and ELOVL4 underlie neurodegeneration in frontotemporal dementia | Scientific Reports, 11(1), Article 21348. | Case control |
| 3. Epidemiology and outcomes | Etiology and risk factors | 2021 | India | Ghosh et al. | Superficial siderosis due to multiple cavernomas: An uncommon cause of early-onset dementia | Psychogeriatrics, 21(3), 434–437. | Case report/series |
| 3. Epidemiology and outcomes | Etiology and risk factors | 2021 | Taiwan | Cheng et al. | Risk of early-onset dementia among persons with tinnitus: a retrospective case–control study | Scientific Reports, 11(1), Article 13399. | Case control |
| 3. Epidemiology and outcomes | Etiology and risk factors | 2022 | South Korea | Kim et al. | Atypical young-onset dementia in cerebral thromboangiitis obliterans: A case report | Alzheimer disease and associated disorders, 36(2), 168–172. | Case report/series |
| 3. Epidemiology and outcomes | Etiology and risk factors | 2022 | Taiwan | Kuang et al. | Absence of an association between macular degeneration and young-onset dementia | Journal of Personalized Medicine, 12(2), 291. | Case control |
| 3. Epidemiology and outcomes | Etiology and risk factors | 2023 | Japan | Shiraki et al. | Influence of age on associations of occlusal status and number of present teeth with dementia in community-dwelling older people in Japan: Cross-sectional study | International Journal of Environmental Research and Public Health, 20(9), 5695. | Cross-sectional/Survey |
| 3. Epidemiology and outcomes | Etiology and risk factors | 2023 | Japan | Hamada et al. | Genetic Creutzfeldt‒Jakob disease with 5-octapeptide repeats presented as frontotemporal dementia | Human Genome Variation, 10(1), Article 10. | Case report/series |
| 3. Epidemiology and outcomes | Etiology and risk factors | 2024 | Japan | Fujii et al. | A case of folate deficiency showing behavioural variant of frontotemporal dementia-like symptoms | Psychogeriatrics, 24(2), 513–517. | Case report/series |
| 3. Epidemiology and outcomes | Etiology and risk factors | 2024 | South Korea | Seo et al. | Association between age at diagnosis of type 2 diabetes and subsequent risk of dementia and its major subtypes | Journal of Clinical Medicine, 13(15), 4386. | Case control |
| 3. Epidemiology and outcomes | Etiology and risk factors | 2024 | South Korea | Park et al. | Association of midlife body-weight variability and cycles with earlier dementia onset: a nationwide cohort study | Alzheimer’s Research & Therapy, 16(1), Article 91. | Cohort |
| 3. Epidemiology and outcomes | Etiology and risk factors | 2024 | South Korea | Heo et al. | Association of remnant cholesterol with risk of dementia: a nationwide population-based cohort study in South Korea | The Lancet. Healthy Longevity, 5(8), e524–e533. | Cohort |
| 3. Epidemiology and outcomes | Etiology and risk factors | 2024 | South Korea | Sohn et al. | Effects of future subjective expectations on cognitive decline and dementia among middle-aged and older adults | Behavioral Sciences, 14(5), 421. | Cohort |
| 3. Epidemiology and outcomes | Etiology and risk factors | 2024 | South Korea | Yoo et al. | Association between depression and young-onset dementia in middle-aged women | Alzheimer’s Research & Therapy, 16(1), Article 137. | Cohort |
| 3. Epidemiology and outcomes | Etiology and risk factors | 2024 | Taiwan | Hung et al. | Association of young-onset dementia with pre-existing peripheral vestibular disorders | Journal of Alzheimer’s Disease, 101(2), 603–610. | Case control |
| 3. Epidemiology and outcomes | Health economics | 2016 | Singapore | Kandiah et al. | Cost related to dementia in the young and the impact of etiological subtype on cost | Journal of Alzheimer’s Disease, 49(2), 277–285. | Cross-sectional/Survey |
| 3. Epidemiology and outcomes | Incidence/prevalence | 1999 | Japan | Hatada et al. | Further evidence of westernization of dementia prevalence in Nagasaki, Japan, and family recognition | International Psychogeriatrics, 11(2), 123–138. | Cohort |
| 3. Epidemiology and outcomes | Incidence/prevalence | 2009 | Japan | Ikejima et al. | Prevalence and causes of early-onset dementia in Japan: a population-based study. | Stroke (1970), 40(8), 2709–2714. | Cohort |
| 3. Epidemiology and outcomes | Incidence/prevalence | 2012 | Japan | Wada-Isoe et al. | Epidemiological survey of frontotemporal lobar degeneration in Tottori prefecture, Japan. | Dementia and Geriatric Cognitive Disorders Extra, 2(1), 381–386. | Cohort |
| 3. Epidemiology and outcomes | Incidence/prevalence | 2014 | Australia | Li et al. | Dementia prevalence and incidence among the indigenous and non-indigenous populations of the northern territory | Medical Journal of Australia, 200(8), 465–469. | Cohort |
| 3. Epidemiology and outcomes | Incidence/prevalence | 2014 | Australia | Withall et al. | The prevalence and causes of younger onset dementia in Eastern Sydney, Australia | International Psychogeriatrics, 26(12), 1955–1965. | Cohort |
| 3. Epidemiology and outcomes | Incidence/prevalence | 2014 | Japan | Ikejima et al. | Multicenter population-based study on the prevalence of early onset dementia in Japan: Vascular dementia as its prominent cause | Psychiatry and Clinical Neurosciences, 68(3), 216–224. | Cohort |
| 3. Epidemiology and outcomes | Incidence/prevalence | 2020 | Japan | Awata et al. | Prevalence and subtype distribution of early-onset dementia in Japan | Psychogeriatrics, 20(6), 817–823. | Cohort |
| 3. Epidemiology and outcomes | Incidence/prevalence | 2020 | Japan | Edahiro et al. | Incidence and distribution of subtypes of early-onset dementia in Japan: A nationwide analysis based on annual performance reports of the Medical Centers for Dementia | Geriatrics & Gerontology International, 20(11), 1050–1055. | Cohort |
| 3. Epidemiology and outcomes | Incidence/prevalence | 2021 | Taiwan | Chang et al. | Age-stratified risk of dementia in Parkinson's disease: A nationwide, population-based, retrospective cohort study in Taiwan | Frontiers in Neurology, 12, 748096. | Cohort |
| 3. Epidemiology and outcomes | Incidence/prevalence | 2022 | New Zealand | Fonseka et al. | Incidence of young onset dementia in Waikato, New Zealand: A population-based study | Journal of Alzheimer’s Disease, 90(3), 1321–1327. | Cohort |
| 3. Epidemiology and outcomes | Incidence/prevalence | 2022 | New Zealand | Ryan et al. | Prevalence of young-onset dementia: Nationwide analysis of routinely collected data | Journal of Neurology, Neurosurgery and Psychiatry, 93(10), 1066–1073. | Cohort |
| 3. Epidemiology and outcomes | Incidence/prevalence | 2023 | South Korea | Hwangbo et al. | Dementia incidence and population-attributable fraction for dementia risk factors in Republic of Korea: A 12-year longitudinal follow-up study of a national cohort | Frontiers in Aging Neuroscience, 15, 1126587. | Cohort |
| 3. Epidemiology and outcomes | Incidence/prevalence | 2024 | Australia | Draper et al. | Young onset dementia in New South Wales, Australia in 1891: What has changed since then? | International Journal of Geriatric Psychiatry, 39(9), e6154. | Cohort |
| 3. Epidemiology and outcomes | Mortality | 2001 | Japan | Ueki et al. | Factors associated with mortality in patients with early-onset Alzheimer's disease: a five-year longitudinal study | International Journal of Geriatric Psychiatry, 16(8), 810–815. | Cohort |
| 3. Epidemiology and outcomes | Mortality | 2013 | South Korea | Go et al. | Survival of Alzheimer's disease patients in Korea | Dementia and Geriatric Cognitive Disorders, 35(3–4), 219–228. | Cohort |
| 3. Epidemiology and outcomes | Mortality | 2017 | South Korea | Chang et al. | Mortality risk after diagnosis of early-onset Alzheimer's disease versus late-onset Alzheimer's disease: A propensity score matching analysis | Journal of Alzheimer’s Disease, 56(4), 1341–1348. | Cross-sectional/Survey |
| 3. Epidemiology and outcomes | Mortality | 2020 | Taiwan | Yeh et al. | Estimating life expectancy and lifetime healthcare costs for Alzheimer's disease in Taiwan: Does the age of disease onset matter? | Journal of Alzheimer’s Disease, 73(1), 307–315. | Cohort |
| 3. Epidemiology and outcomes | Mortality | 2021 | Australia | Sexton et al. | Suicide in frontotemporal dementia and Huntington disease: analysis of family-reported pedigree data and implications for genetic healthcare for asymptomatic relatives | Psychology & Health, 36(11), 1397–1402. | Cross-sectional/Survey |
| 3. Epidemiology and outcomes | Mortality | 2022 | Australia | Loi et al. | Mortality in dementia is predicted by older age of onset and cognitive presentation | Australian and New Zealand Journal of Psychiatry, 56(7), 852–861. | Cohort |
| 3. Epidemiology and outcomes | Mortality | 2022 | Australia | Loi et al. | Risk factors to mortality and causes of death in frontotemporal dementia: An Australian perspective | International Journal of Geriatric Psychiatry, 37(2). | Cohort |
| 3. Epidemiology and outcomes | Mortality | 2022 | South Korea | Park et al. | Effects of socioeconomic status and residence areas on long-term survival in patients with early-onset dementia: The Korean national health insurance service database study | Journal of Korean Medical Science, 37(49), e354. | Cohort |
| 3. Epidemiology and outcomes | Mortality | 2023 | Australia | Loi et al. | Survival in Huntington’s disease and other young-onset dementias | International Journal of Geriatric Psychiatry, 38(4), e5913. | Cohort |
| 3. Epidemiology and outcomes | Mortality | 2023 | Australia | Yoo et al. | Comparing survival and mortality in patients with late-onset and young-onset vascular dementia | International Psychogeriatrics, 35(9), 519–527. | Cohort |
| 3. Epidemiology and outcomes | Mortality | 2023 | Taiwan | Liu et al. | Risks of organ failures and deaths associated with young-onset dementia after hospitalizations for motor vehicle crash injuries: a nationwide population-based retrospective cohort study | Annals of Neurology, 48(2), 194–200. | Case control |
| 4. Neuropathology | Neuropathology | 1976 | Japan | Kosaka et al. | Presenile dementia with Alzheimer-, Pick- and Lewy-body changes | Acta Neuropathologica, 36(3), 221–233. | Case report/series |
| 4. Neuropathology | Neuropathology | 1980 | Japan | Yagishita et al. | The fine structure of neurofibrillary tangles in a case of atypical presenile dementia | Journal of the Neurological Sciences, 48(3), 325–332. | Case report/series |
| 4. Neuropathology | Neuropathology | 1985 | Japan | Mitsuyama et al. | Progressive dementia with motor neuron disease An additional case report and neuropathological review of 20 cases in Japan | European Archives of Psychiatry and Neurological Sciences, 235(1), 1–8. | Case report/series |
| 4. Neuropathology | Neuropathology | 1986 | Japan | Sasaki et al. | Regional distribution of amino acid transmitters in postmortem brains of presenile and senile dementia of Alzheimer type | Annals of Neurology, 19(3), 263–269. | Case control |
| 4. Neuropathology | Neuropathology | 1986 | Japan | Shibayama et al. | Unusual cases of presenile dementia with Fahr's syndrome | Japanese Journal of Psychiatry and Neurology, 40(1), 85–100. | Case report/series |
| 4. Neuropathology | Neuropathology | 1989 | Japan | Fukutani et al. | An autopsy case of familial juvenile Alzheimer's disease with extensive involvement of the subcortical gray and white matters | Acta Neuropathologica, 77(3), 329–332. | Case report/series |
| 4. Neuropathology | Neuropathology | 1990 | Japan | Tsuchiya et al. | Neuropathological study of the amygdala in presenile Alzheimer's disease | J. Neurosci, 100(1), 165–173. | Cross-sectional/Survey |
| 4. Neuropathology | Neuropathology | 1992 | Australia | Crowe et al. | Diffuse lewy body disease and progressive dementia in a young woman | Australian and New Zealand journal of psychiatry, 26(3), 507–511. | Case report/series |
| 4. Neuropathology | Neuropathology | 1992 | Japan | Arima et al. | Presenile dementia with progressive supranuclear palsy tangles and Pick bodies: an unusual degenerative disorder involving the cerebral cortex, cerebral nuclei, and brain stem nuclei | Acta Neuropathologica, 84(2), 128–134. | Case report/series |
| 4. Neuropathology | Neuropathology | 1992 | Japan | Okamoto et al. | Ubiquitin-positive intraneuronal inclusions in the extramotor cortices of presenile dementia patients with motor neuron disease | Journal of Neurology, 239(8), 426–430. | Cross-sectional/Survey |
| 4. Neuropathology | Neuropathology | 1994 | Japan | Iijirna et al. | A case of presenile dementia with neurofibrillary tangles but without senile plaques in small neurons of the external granular layer of the cerebral cortex | Neuropathology, 14(1), 57–65. | Case report/series |
| 4. Neuropathology | neuropathology | 1994 | Japan | Kosaka | Diffuse neurofibrillary tangles with calcification: A new presenile dementia | Journal of Neurology, Neurosurgery and Psychiatry, 57(5), 594–596. | Case report/series |
| 4. Neuropathology | Neuropathology | 1995 | Japan | Nagaoka et al. | A juvenile case of frontotemporal dementia: Neurochemical and neuropathological investigation | Progress in neuro-psychopharmacology & biological psychiatry, 19(8), 1251–1261. | Case report/series |
| 4. Neuropathology | Neuropathology | 1997 | Japan | Arima et al. | Argyrophilic tan-positive twisted and non-twisted tubules in astrocytic processes in brains of Alzheimer-type dementia: An electron microscopical study | Acta Neuropathologica, 95(1), 28–39. | Cross-sectional/Survey |
| 4. Neuropathology | Neuropathology | 1998 | Japan | Mimura et al. | Presenile non-Alzheimer dementia with motor neuron disease and laminar spongiform degeneration | Neuropathology, 18(1), 19–26. | Case report/series |
| 4. Neuropathology | Neuropathology | 2000 | Japan | Ikeda | Neuropathological discrepancy between Japanese Pick's disease without Pick bodies and frontal lobe degeneration type of frontotemporal dementia proposed by Lund and Manchester Group | Neuropathology, 20(1), 76–82. | Cross-sectional/Survey |
| 4. Neuropathology | Neuropathology | 2000 | Japan | Tanabe et al. | Tau pathology in diffuse neurofibrillary tangles with calcification (DNTC): Biochemical and immunohistochemical investigation | Neuroreport, 11(11), 2473–2476. | Cross-sectional/Survey |
| 4. Neuropathology | Neuropathology | 2001 | Japan | Mimura et al. | Corticobasal degeneration presenting with nonfluent primary progressive aphasia: A clinicopathological study | Journal of the Neurological Sciences, 183(1), 19–26. | Case report/series |
| 4. Neuropathology | Neuropathology | 2002 | Japan | Umahara et al. | Demonstration and distribution of tau-positive glial coiled body-like structures in white matter and white matter threads in early onset Alzheimer's disease | Neuropathology, 22(1), 9–12. | Case report/series |
| 4. Neuropathology | Neuropathology | 2003 | Japan | Yokota et al. | Variability and heterogeneity in Alzheimer's disease with cotton wool plaques: A clinicopathological study of four autopsy cases | Acta Neuropathologica, 106(4), 348–356. | Case report/series |
| 4. Neuropathology | Neuropathology | 2005 | Japan | Ishihara et al. | Argyrophilic grain disease presenting with frontotemporal dementia: A neuropsychological and pathological study of an autopsied case with presenile onset | Neuropathology, 25(2), 165–170. | Case report/series |
| 4. Neuropathology | Neuropathology | 2005 | Japan | Shibuya-Tayoshi et al. | Presenile dementia mimicking Pick's disease: An autopsy case of localized amygdala degeneration with character change and emotional disorder | Neuropathology, 25(3), 235–240. | Case report/series |
| 4. Neuropathology | Neuropathology | 2006 | Japan | Ishihara et al. | An autopsy case of frontotemporal dementia with severe dysarthria and motor neuron disease showing numerous basophilic inclusions | Neuropathology, 26(5), 447–454. | Case report/series |
| 4. Neuropathology | Neuropathology | 2007 | Japan | Yokota et al. | Lewy body variant of Alzheimer's disease or cerebral type Lewy body disease? Two autopsy cases of presenile onset with minimal involvement of the brainstem | Neuropathology, 27(1), 21–35. | Case report/series |
| 4. Neuropathology | Neuropathology | 2008 | Australia | Bermingham et al. | Frontotemporal dementia and Parkinsonism linked to chromosome 17 in a young Australian patient with the G389R Tau mutation. | Neuropathology and Applied Neurobiology, 34(3), 366–370. | Case report/series |
| 4. Neuropathology | Neuropathology | 2010 | Japan | Kuwahara et al. | Frontotemporal lobar degeneration with motor neuron disease showing severe and circumscribed atrophy of anterior temporal lobes | Journal of the Neurological Sciences, 297(1), 92–96. | Case report/series |
| 4. Neuropathology | Neuropathology | 2010 | Japan | Mimuro et al. | Neuronal and glial tau pathology in early frontotemporal lobar degeneration-tau, Pick's disease subtype | Journal of the Neurological Sciences, 290(1), 177–182. | Case report/series |
| 4. Neuropathology | neuropathology | 2010 | Japan | Okazaki et al. | Alzheimer's disease: Report of two autopsy cases with a clinical diagnosis of corticobasal degeneration | Neuropathology, 30(2), 140–148. | Case report/series |
| 4. Neuropathology | Neuropathology | 2011 | Japan | Aoki et al. | Gray matter lesions in Nasu-Hakola disease: a report on three autopsy cases | Neuropathology, 31(2), 135–143. | Case report/series |
| 4. Neuropathology | Neuropathology | 2013 | South Korea | Cho et al. | Amyloid deposition in early onset versus late onset Alzheimer's disease | Journal of Alzheimer’s Disease, 35(4), 813–821. | Case control |
| 4. Neuropathology | Neuropathology | 2016 | Japan | Matsumoto et al. | An autopsy case of frontotemporal lobar degeneration with the appearance of fused in sarcoma inclusions (basophilic inclusion body disease) clinically presenting corticobasal syndrome | Neuropathology, 36(1), 77–87. | Case report/series |
| 4. Neuropathology | Neuropathology | 2017 | Australia | Bradfield et al. | Rapidly progressive Fronto-temporal dementia (FTD) associated with Frontotemporal lobar degeneration (FTLD) in the presence of Fused in Sarcoma (FUS) protein: A rare, sporadic, and aggressive form of FTD | International Psychogeriatrics, 29(10), 1743–1746. | Case report/series |
| 4. Neuropathology | Neuropathology | 2017 | Japan | Ikeda et al. | Pick's disease with neuronal four-repeat tau accumulation in the basal ganglia, brain stem nuclei and cerebellum | Neuropathology, 37(6), 544–559. | Case report/series |
| 4. Neuropathology | Neuropathology | 2017 | Japan | Iwasaki et al. | An autopsied case of corticobasal degeneration presenting with frontotemporal dementia followed by myoclonus | Neuropathology, 37(6), 569–574 | Case report/series |
| 4. Neuropathology | Neuropathology | 2018 | Australia | Tan et al. | Multiple neuronal pathologies are common in young patients with pathologically proven Frontotemporal lobar degeneration | Neuropathology and Applied Neurobiology, 44(5), 522–532. | Cross-sectional/Survey |
| 4. Neuropathology | Neuropathology | 2018 | Japan | Hirano et al. | Voxel-based acetylcholinesterase PET study in early and late onset Alzheimer's disease | Journal of Alzheimer’s Disease, 62(4), 1539–1548. | Case control |
| 4. Neuropathology | Neuropathology | 2020 | Japan | Uchida et al. | Corticobasal syndrome-Pick's disease: A clinicopathological study | Journal of the Neurological Sciences, 412, Article 116752. | Case report/series |
| 4. Neuropathology | Neuropathology | 2021 | Japan | Kawakatsu et al. | Clinicopathological heterogeneity of Alzheimer's disease with pure Alzheimer's disease pathology: Cases associated with dementia with Lewy bodies, very early-onset dementia, and primary progressive aphasia | Neuropathology, 41(6), 427–449. | Case report/series |
| 4. Neuropathology | neuropathology | 2022 | Japan | Beck et al. | An autopsy case of Alzheimer's disease with amygdala-predominant Lewy pathology presenting with frontotemporal dementia-like psychiatric symptoms | Neuropathology, 42(2), 147–154. | Case report/series |
| 4. Neuropathology | Neuropathology | 2022 | Japan | Kobayashi et al. | Limbic-predominant age-related TDP-43 encephalopathy characterised by frontotemporal dementia-like behavioural symptoms | Psychogeriatrics, 22(4), 574–579. | Case report/series |
| 4. Neuropathology | Neuropathology | 2023 | Australia | Panegyres et al. | Brain amyloid in sporadic young onset Alzheimer's disease | JAD Reports, 7(1), 263–270. | Case control |
| 4. Neuropathology | Neuropathology | 2023 | China | Lv et al. | High burdens of phosphorylated tau protein and distinct precuneus atrophy in sporadic early-onset Alzheimer's disease | Science Bulletin, 68(22), 2817–2826. | Cohort |
| 4. Neuropathology | Neuropathology | 2023 | China | Lu et al. | The heterogeneity of asymmetric tau distribution is associated with an early age at onset and poor prognosis in Alzheimer's disease | NeuroImage Clinical, 38, Article 103416. | Cross-sectional/Survey |
| 5. Psychosocial impacts | Carers and families | 2005 | Japan | Takano et al. | Gender difference and caregivers' burden in early-onset Alzheimer's disease | Psychogeriatrics, 5(3), 73–77. | Cross-sectional/Survey |
| 5. Psychosocial impacts | Carers and families | 2006 | Australia | Kaiser et al. | The psychosocial impact of young onset dementia on spouses | American Journal of Alzheimer’s Disease and Other Dementias, 21(6), 398–402. | Cross-sectional/Survey |
| 5. Psychosocial impacts | Carers and families | 2007 | Japan | Arai et al. | Do family caregivers perceive more difficulty when they look after patients with early onset dementia compared to those with late onset dementia? | International Journal of Geriatric Psychiatry, 22, 1255–1261. | Cross-sectional/Survey |
| 5. Psychosocial impacts | Carers and families | 2014 | Taiwan | Lin et al. | Primary caregivers' awareness and perception of early-onset dementia conditions in adolescents and young and middle-aged adults with Down syndrome | Research in Developmental Disabilities, 35(9), 1934–1940. | Cross-sectional/Survey |
| 5. Psychosocial impacts | Carers and families | 2016 | Australia | Hutchinson et al. | Empowerment of young people who have a parent living with dementia: A social model perspective | International Psychogeriatrics, 28(4), 657–668. | Qualitative |
| 5. Psychosocial impacts | Carers and families | 2016 | Australia | Hutchinson et al. | The emotional well-being of young people having a parent with younger onset dementia | Dementia (London, England), 15(4), 609–628. | Qualitative |
| 5. Psychosocial impacts | Carers and families | 2017 | Singapore | Lim et al. | High caregiver burden in young onset dementia: What factors need attention? | Journal of Alzheimer’s Disease, 61(2), 537–543. | Cross-sectional/Survey |
| 5. Psychosocial impacts | Carers and families | 2019 | Hong Kong | Pang et al. | Finding positives in caregiving: The unique experiences of Chinese spousal caregivers of persons with young-onset dementia | Dementia (London, England), 18(5), 1615–1628. | Qualitative |
| 5. Psychosocial impacts | Carers and families | 2020 | Singapore | Wang et al. | Does health-related quality of life in Asian informal caregivers differ between early-onset dementia and late-onset dementia? | Psychogeriatrics, 20(5), 608–619. | Cross-sectional/Survey |
| 5. Psychosocial impacts | Carers and families | 2021 | Australia | Claire et al. | Supporting behaviour change in younger-onset dementia: mapping the needs of family carers in the community | Aging & Mental Health, 26(11), 2252–2261. | Cross-sectional/Survey |
| 5. Psychosocial impacts | Carers and families | 2022 | Australia | Kang et al. | Carer burden and psychological distress in young-onset dementia: An Australian perspective | International Journal of Geriatric Psychiatry, 37(7). | Cross-sectional/Survey |
| 5. Psychosocial impacts | carers and families | 2022 | China | Kang et al. | Qualitative study on the dyadic experience of patients with young-onset dementia and their caregivers [年轻型痴呆症患者及其照护者疾病体验的质性研究] | Chinese Journal of Nursing, 57(13): 1591-1598. | Qualitative |
| 5. Psychosocial impacts | Carers and families | 2023 | Australia | Kang et al. | Carer burden and behavioral disturbance is similar between younger-onset alzheimer's disease and behavioral variant frontotemporal dementia | International Psychogeriatrics, 36(6), 474–481. | Cross-sectional/Survey |
| 5. Psychosocial impacts | Carers and families | 2023 | Australia | Perin et al. | Online counselling for family carers of people with young onset dementia: The RHAPSODY-Plus pilot study | Digital Health, 9, 20552076231161962. | Mixed-methods |
| 5. Psychosocial impacts | Carers and families | 2023 | Australia | Poulton et al. | Family communication about diagnostic genetic testing for younger-onset dementia | Journal of Personalized Medicine, 13(4), 621. | Qualitative |
| 5. Psychosocial impacts | Carers and families | 2024 | China | Cui et al. | Navigating the journey of living with young-onset dementia: Experiences of spousal caregivers | Journal of Alzheimer’s Disease, 101(1), 197–209. | Qualitative |
| 5. Psychosocial impacts | Carers and families | 2024 | South Korea | Yang et al. | Understanding family resilience in young-onset dementia: A multiple case study | Journal of Advanced Nursing, 81(7), 4023–4037. | Qualitative |
| 5. Psychosocial impacts | Driving | 2022 | Taiwan | Liu et al. | Association between young-onset dementia and risk of hospitalization for motor vehicle crash injury in Taiwan | JAMA Network Open, 5(5), e2210474. | Case control |
| 5. Psychosocial impacts | Driving | 2023 | Australia | Scott et al. | A qualitative study exploring the experiences and needs of people living with young onset dementia related to driving cessation: 'It's like you get your legs cut off' | Age and Ageing, 52(7) | Qualitative |
| 5. Psychosocial impacts | Employment | 2013 | Australia | Robertson et al. | Side by Side: A workplace engagement program for people with younger onset dementia | Dementia (London, England), 12(5), 666–674. | Qualitative |
| 5. Psychosocial impacts | Employment | 2015 | Australia | Robertson et al. | Evaluation of a workplace engagement project for people with younger onset dementia | Journal of Clinical Nursing, 24(15–16), 2331–2339. | Qualitative |
| 5. Psychosocial impacts | Employment | 2017 | Japan | Sakata et al. | Job loss after diagnosis of early-onset dementia: A matched cohort study | Journal of Alzheimer’s Disease, 60(4), 1231–1235. | Case control |
| 5. Psychosocial impacts | Employment | 2019 | Australia | Evans | An exploration of the impact of younger-onset dementia on employment | Dementia (London, England), 18(1), 262–281. | Qualitative |
| 5. Psychosocial impacts | Employment | 2020 | Japan | Omote et al. | Investigation into the factors relating to the intention of workplaces to retain employees diagnosed with Young Onset Dementia. | Journal of wellness and health care 44 (1), 43-52. | Qualitative |
| 5. Psychosocial impacts | Employment | 2022 | Japan | Ikeuchi et al. | Work-related experiences of people living with young-onset dementia in Japan | Health & Social Care in the Community, 30(2), 548–557. | Qualitative |
| 5. Psychosocial impacts | Employment | 2023 | Japan | Omote et al. | Experience with support at workplaces for people with young onset dementia: A qualitative evaluation of being open about dementia | International Journal of Environmental Research and Public Health, 20(13), 6235. | Qualitative |
| 6. Support, intervention and services | Support, intervention and services | 1998 | Australia | Luscombe et al. | Younger people with dementia: Diagnostic issues, effects on carers and use of services | International Journal of Geriatric Psychiatry, 13(5), 323–330. | Cross-sectional/Survey |
| 6. Support, intervention and services | Support, intervention and services | 2012 | Japan | Yokokawa | Usefulness of video for observing lifestyle impairments in dementia patients | Psychogeriatrics, 12(2), 137–141. | Case report/series |
| 6. Support, intervention and services | Support, intervention and services | 2013 | Australia | Armari et al. | The needs of patients with early onset dementia | American Journal of Alzheimer’s Disease and Other Dementias, 28(1), 42–46. | Cross-sectional/Survey |
| 6. Support, intervention and services | Support, intervention and services | 2015 | Taiwan | Lin et al. | Demographic and medication characteristics of traditional Chinese medicine users among dementia patients in Taiwan: A nationwide database study | Journal of Ethnopharmacology, 161(NA), 108–115. | Case control |
| 6. Support, intervention and services | Support, intervention and services | 2017 | Australia | Cations et al. | Why aren’t people with young onset dementia and their supporters using formal services? Results from the INSPIRED study | PloS One, 12(7), e0180935. | Mixed-methods |
| 6. Support, intervention and services | Support, intervention and services | 2017 | New Zealand | Rimkeit et al. | Experiencing place: Younger people with dementia facing aged care | Australasian Psychiatry : Bulletin of the Royal Australian and New Zealand College of Psychiatrists, 25(6), 554–561. | Qualitative |
| 6. Support, intervention and services | Support, intervention and services | 2020 | Australia | Vafeas et al. | A younger onset dementia toolkit: Innovative practice | Dementia (London, England), 19(4), 1299–1307. | Experimental |
| 6. Support, intervention and services | Support, intervention and services | 2020 | Australia | Hutchinson et al. | Co-creation of a family-focused service model living with younger onset dementia | Dementia (London, England), 19(4), 1029–1050. | Qualitative |
| 6. Support, intervention and services | Support, intervention and services | 2021 | Japan | Omata et al. | Collaborative development of outing assistants for people with dementia: A case study on a co-design approach | Asian CHI Symposium 2021, 21-24. | Qualitative |
| 6. Support, intervention and services | Support, intervention and services | 2021 | South Korea | Jeon et al. | Impact of mixed cognitive intervention training on early onset dementia | Osong Public Health and Research Perspectives, 12(1), 29–36. | Experimental |
| 6. Support, intervention and services | Support, intervention and services | 2021 | Taiwan | Tu et al. | A concept of mobile support system for the young onset dementia in Taiwan | Studies in health technology and informatics, 284, 442–443. | Cross-sectional/Survey |
| 6. Support, intervention and services | Support, intervention and services | 2022 | Australia | Loi et al. | Music and psychology & social connections program: Protocol for a novel intervention for dyads affected by younger-onset dementia | Brain Sciences, 12(4), 503. | Experimental |
| 6. Support, intervention and services | Support, intervention and services | 2022 | Australia | Cations et al. | Post-diagnosis young-onset dementia care in the National Disability Insurance Scheme | Australian and New Zealand Journal of Psychiatry, 56(3), 270–280. | Cohort |
| 6. Support, intervention and services | Support, intervention and services | 2022 | Australia | Couzner et al. | What do health professionals need to know about young onset dementia? An international Delphi consensus study | BMC Health Services Research, 22(1), Article 14. | Cross-sectional/Survey |
| 6. Support, intervention and services | Support, intervention and services | 2022 | Australia | Day et al. | Cross-sector learning collaboratives can improve post-diagnosis care integration for people with young onset dementia | Health & Social Care in the Community, 30(6), e6135–e6144. | Mixed-methods |
| 6. Support, intervention and services | Support, intervention and services | 2022 | Japan | Nagata et al. | Usefulness of an online system to support daily life activities of outpatients with young-onset dementia: a case report | Psychogeriatrics, 22(6), 890–894. | Case report/series |
| 6. Support, intervention and services | Support, intervention and services | 2023 | Australia | Cadwallader et al. | Post-diagnostic support for behaviour changes in young-onset dementia in Australia | Brain Sciences, 13(11), 1529. | Cross-sectional/Survey |
| 6. Support, intervention and services | Support, intervention and services | 2023 | Australia | Marks et al. | Assistance dogs for people with younger (early)-onset dementia: The family carer’s experience | Animals (Basel), 13(5), 777. | Qualitative |
| 6. Support, intervention and services | Support, intervention and services | 2023 | Australia | Brown et al. | Understanding clinician's experiences with implementation of a younger onset dementia telehealth service | Journal of Geriatric Psychiatry and Neurology, 36(4), 295–308. | Qualitative |
| 6. Support, intervention and services | Support, intervention and services | 2024 | Australia | Loi et al. | Referral reasons to a Victorian mental health triage service for individuals living with young-onset dementia | Australian Health Review, 48(4), 358–363. | Cross-sectional/Survey |
| 6. Support, intervention and services | Support, intervention and services | 2024 | Australia | Atee et al. | Behaviours and psychological symptoms of childhood dementia: two cases of psychosocial interventions | Palliative Care and Social Practice, 18, 26323524241273492. | Case report/series |
| 6. Support, intervention and services | Support, intervention and services | 2024 | Australia | Loi et al. | A mixed methods evaluation of the music and psychology and social connections (MAPS) pilot – A dyadic intervention for couples affected by young-onset dementia | Dementia (London, England), 23(7), 1103–1125. | Mixed-methods |
| 6. Support, intervention and services | Support, intervention and services | 2024 | Australia | Loi et al. | Younger people with dementia registered to public mental health services in Victoria, Australia | Australian Health Review, 48(4), 351–357. | Cross-sectional/Survey |
| 6. Support, intervention and services | Support, intervention and services | 2024 | Japan | Kogata et al. | An analysis of the contents of the young-onset dementia helpline: profiles of clients who consulted the helpline themselves | Psychogeriatrics, 24(3), 617–626. | Cross-sectional/Survey |
| 7. Diagnosis and assessment | Diagnosis and assessment | 2016 | Australia | Draper et al. | Time to diagnosis in young-onset dementia and its determinants: the INSPIRED study | International Journal of Geriatric Psychiatry, 31(11), 1217–1224. | Cross-sectional/Survey |
| 7. Diagnosis and assessment | Diagnosis and assessment | 2020 | South Korea | Kim et al. | A comprehensive evaluation of the process of copying a complex figure in early- And late-onset alzheimer disease: A quantitative analysis of digital pen data | Journal of Medical Internet Research, 22(8), e18136. | Cross-sectional/Survey |
| 7. Diagnosis and assessment | Diagnosis and assessment | 2022 | Australia | Loi et al. | Time to diagnosis in younger-onset dementia and the impact of a specialist diagnostic service | International Psychogeriatrics, 34(4), 367–375. | Cross-sectional/Survey |
| 7. Diagnosis and assessment | Diagnosis and assessment | 2022 | India | Ellajosyula et al. | Delay in the diagnosis of dementia in urban India: Role of dementia subtype and age at onset | International Journal of Geriatric Psychiatry, 37(12). | Cross-sectional/Survey |
| 7. Diagnosis and assessment | Diagnosis and assessment | 2023 | Australia | Lai et al. | Journey to diagnosis of young-onset dementia: A qualitative study of people with young-onset dementia and their family caregivers in Australia | Dementia (London, England), 22(5), 1097–1114. | Qualitative |
| 7. Diagnosis and assessment | Diagnosis and assessment | 2023 | Australia | Burkinshaw et al. | System and policy-level barriers and facilitators for timely and accurate diagnosis of young onset dementia | International Journal of Geriatric Psychiatry, 38(1), e5859-n/a. | Qualitative |
| 7. Diagnosis and assessment | Diagnosis and assessment | 2024 | Australia | Brown et al. | Investigating equivalence of in-person and telehealth-based neuropsychological assessment performance for individuals being investigated for younger onset dementia | Archives of Clinical Neuropsychology, 39(5), 594–607. | Diagnostic accuracy |
| 7. Diagnosis and assessment | Diagnosis and assessment | 2024 | Japan | Taomoto et al. | Utility of the Japanese version of the Clinical Dementia Rating® plus National Alzheimer's Coordinating Centre Behaviour and Language Domains for sporadic cases of frontotemporal dementia in Japan | Psychogeriatrics, 24(2), 281–294. | Cohort |

**Supplementary table S2:** Research topics of the 437 empirical Asia-Pacific studies by countries/territories

| Research Topics | Australia | China | Hong Kong | India | Japan | Malaysia | New Zealand | Philippines | Singapore | South Korea | Sri Lanka | Sri Lanka + India | Taiwan | Thailand | Vietnam | Grand  Total |
| --- | --- | --- | --- | --- | --- | --- | --- | --- | --- | --- | --- | --- | --- | --- | --- | --- |
| **1. Biological and neuroimaging investigations** | **12** | **47** |  | **6** | **66** | **3** | **1** | **1** | **5** | **37** |  | **1** | **5** | **3** | **2** | **189** |
| Genetics | 7 | 35 |  | 3 | 38 | 3 | 1 | 1 | 1 | 23 |  | 1 | 1 | 3 | 1 | 118 |
| Neuroimaging | 2 | 8 |  | 2 | 16 |  |  |  |  | 11 |  |  | 3 |  | 1 | 43 |
| Cerebrospinal fluid | 2 | 1 |  |  | 7 |  |  |  | 3 |  |  |  |  |  |  | 13 |
| Electroencephalogram | 1 | 1 |  |  | 4 |  |  |  |  |  |  |  |  |  |  | 6 |
| Blood |  |  |  |  |  |  |  |  |  | 1 |  |  | 1 |  |  | 2 |
| Multimodal investigations |  | 2 |  | 1 | 1 |  |  |  | 1 | 2 |  |  |  |  |  | 7 |
| **2. Clinical features and course** | **16** | **12** | **4** | **12** | **27** | **1** | **2** |  | **2** | **10** | **1** |  | **4** | **2** |  | **93** |
| **3. Epidemiology and outcomes** | **13** |  |  | **4** | **13** |  | **2** |  | **1** | **14** |  |  | **7** |  |  | **54** |
| Etiology and risk factors | 4 |  |  | 4 | 6 |  |  |  |  | 8 |  |  | 4 |  |  | 26 |
| Incidence/prevalence | 3 |  |  |  | 6 |  | 2 |  |  | 1 |  |  | 1 |  |  | 13 |
| Mortality | 5 |  |  |  | 1 |  |  |  |  | 3 |  |  | 2 |  |  | 11 |
| Health economics |  |  |  |  |  |  |  |  | 1 |  |  |  |  |  |  | 1 |
| Combined epidemiological studies | 1 |  |  |  |  |  |  |  |  | 2 |  |  |  |  |  | 3 |
| **4. Neuropathology** | **5** | **2** |  |  | **35** |  |  |  |  | **1** |  |  |  |  |  | **43** |
| **5. Psychosocial impacts** | **12** | **2** | **1** |  | **6** |  |  |  | **2** | **1** |  |  | **2** |  |  | **26** |
| Carers and families | 8 | 2 | 1 |  | 2 |  |  |  | 2 | 1 |  |  | 1 |  |  | 17 |
| Employment | 3 |  |  |  | 4 |  |  |  |  |  |  |  |  |  |  | 7 |
| Driving | 1 |  |  |  |  |  |  |  |  |  |  |  | 1 |  |  | 2 |
| **6. Support, intervention and services** | **16** |  |  |  | **4** |  | **1** |  |  | **1** |  |  | **2** |  |  | **24** |
| **7. Diagnosis and assessment** | **5** |  |  | **1** | **1** |  |  |  |  | **1** |  |  |  |  |  | **8** |
| Grand Total | 79 | 63 | 5 | 23 | 152 | 4 | 6 | 1 | 10 | 65 | 1 | 1 | 20 | 5 | 2 | 437 |

**Supplementary table S3:** Study type published by each Asia-Pacific country/territory

|  | Australia | China | Hong Kong | India | Japan | Malaysia | New Zealand | Philippines | Singapore | South Korea | Sri Lanka | Sri Lanka + India | Taiwan | Thailand | Vietnam | Grand Total |
| --- | --- | --- | --- | --- | --- | --- | --- | --- | --- | --- | --- | --- | --- | --- | --- | --- |
| Case report/series | 7 | 27 | 3 | 13 | 70 | 4 | 1 | 1 |  | 21 |  | 1 | 2 | 2 |  | 152 |
| Case control | 11 | 15 |  | 3 | 36 |  |  |  | 3 | 18 | 1 |  | 10 | 1 |  | 98 |
| Cross-sectional/survey | 24 | 12 | 1 | 4 | 31 |  | 1 |  | 3 | 12 |  |  | 4 | 1 | 2 | 95 |
| Cohort | 17 | 7 |  | 3 | 11 |  | 3 |  | 4 | 12 |  |  | 4 | 1 |  | 62 |
| Qualitative | 13 | 2 | 1 |  | 4 |  | 1 |  |  | 1 |  |  |  |  |  | 22 |
| Experimental | 2 |  |  |  |  |  |  |  |  | 1 |  |  |  |  |  | 3 |
| Mixed-methods | 4 |  |  |  |  |  |  |  |  |  |  |  |  |  |  | 4 |
| Diagnostic accuracy | 1 |  |  |  |  |  |  |  |  |  |  |  |  |  |  | 1 |
| **Grand Total** | **79** | **63** | **5** | **23** | **152** | **4** | **6** | **1** | **10** | **65** | **1** | **1** | **20** | **5** | **2** | **437** |
